# A mutation in a splicing factor that causes retinitis pigmentosa has a transcriptome-wide effect on mRNA splicing

**DOI:** 10.1186/1756-0500-7-401

**Published:** 2014-06-27

**Authors:** Paul K Korir, Lisa Roberts, Raj Ramesar, Cathal Seoighe

**Affiliations:** 1 School of Mathematics, Statistics and Applied Mathematics, National University of Ireland, Galway, University Road, Galway, Republic of Ireland; 2 UCT/MRC Human Genetics Research Unit, Division of Human Genetics, Institute for Infectious Diseases and Molecular Medicine, Faculty of Health Sciences, University of Cape Town, Cape Town, South Africa

**Keywords:** mRNA splicing, Retinitis pigmentosa, Exon array, *PRPF8*

## Abstract

**Background:**

Substantial progress has been made in the identification of sequence elements that control mRNA splicing and the genetic variants in these elements that alter mRNA splicing (referred to as splicing quantitative trait loci – sQTLs). Genetic variants that affect mRNA splicing in *trans* are harder to identify because their effects can be more subtle and diffuse, and the variants are not co-located with their targets. We carried out a transcriptome-wide analysis of the effects of a mutation in a ubiquitous splicing factor that causes retinitis pigmentosa (RP) on mRNA splicing, using exon microarrays.

**Results:**

Exon microarray data was generated from whole blood samples obtained from four individuals with a mutation in the splicing factor *PRPF8* and four sibling controls. Although the mutation has no known phenotype in blood, there was evidence of widespread differences in splicing between cases and controls (affecting approximately 20% of exons). Most probesets with significantly different inclusion (defined as the expression intensity of the exon divided by the expression of the corresponding transcript) between cases and controls had higher inclusion in cases and corresponded to exons that were shorter than average, AT rich, located towards the 5’ end of the gene and flanked by long introns. Introns flanking affected probesets were particularly depleted for the shortest category of introns, associated with splicing via intron definition.

**Conclusions:**

Our results show that a mutation in a splicing factor, with a phenotype that is restricted to retinal tissue, acts as a *trans*-sQTL cluster in whole blood samples. Characteristics of the affected exons suggest that they are spliced co-transcriptionally and via exon definition. However, due to the small sample size available for this study, further studies are required to confirm the widespread impact of this *PRPF8* mutation on mRNA splicing outside the retina.

## Background

Splicing sits at the intersection between transcription and translation and directly regulates both the abundance and diversity of transcripts. It is effected by the spliceosome, a macromolecular complex composed of uridine-rich snRNAs (U1, U2, U5, and U4/U6 duplex for the U2-type spliceosome) and numerous proteins [1], which systematically assemble at conserved sequence motifs on newly-transcribed RNA. The spliceosome undergoes a series of structural rearrangements to catalyse two transesterification reactions ligating adjacent exons [2]. The *PRPF8* protein mediates most spliceosomal interactions as it interfaces with splice sites on the transcribed RNA, snRNAs, and other proteins. Over the years, mapping of *PRPF8* protein domains has revealed numerous splicing-related roles. For example, the 3’ fidelity region attenuates the impact of 3’ splice site (3SS) mutations [3]. Also, the C-terminal domain (CTD) of *Prp8p* (yeast homologue of *PRPF8*) interacts with *Snu114p* and *Brr2p* (another protein that interacts with the CTD) to unwind and release the U4 snRNA, which activates the spliceosome [4-8]. Recent evidence suggests a direct role in splicing through catalysis of the transesterification reactions [9]. It is therefore unsurprising that mutations in *PRPF8* have been shown to affect splicing efficiency in yeast [10], mouse [11,12], zebrafish [13] and human [14].

Genome-wide association studies (GWAS) have revealed a large number of genetic variants that are associated with diseases and phenotypes but for many of these associations the exact causal mechanism remains unknown. A large proportion of the associations are likely to be mediated by the effects of polymorphic variants on gene expression. In support of this view, GWAS results have been found to be enriched for expression quantitative trait loci (eQTLs) [15]. Genetic variants may also affect transcript processing. The contribution of variants that affect mRNA splicing, in particular, is thought to be substantial [16,17]. Although many studies have assessed the impact of genetic variants acting in *cis* on mRNA splicing [16,18-22] variants that act in *trans* have been less extensively studied [23].

Genetic variants affecting components of the spliceosome can have *trans*-acting effects on splicing. In particular, maturation defects, structural malformations and splicing factor mutations can lead to aberrant transcripts due to mis-splicing, resulting in genetic diseases [24,25]. For example microencephalic osteodysplastic primordial dwarfism type I (MOPDI), also known as Taybi-Linder Syndrome (TALS), is caused by mutations in the minor spliceosome resulting in incomplete splicing of a small number of critical transcripts [26,27]. MOPDI is characterised by gross developmental retardation and a lifespan of under one year. Mutations affecting components of the spliceosome can also have a more restricted phenotype, such as in the case of splicing factor associated retinitis pigmentosa (RP). RP is a broad spectrum of eye diseases featuring gradual degeneration of rod and cone photoreceptors, causally associated with mutations in over 100 genes and may be autosomal dominant, autosomal recessive or X-linked [28,29]. A subset of autosomal dominant mutations are located on components of the spliceosome *PRPF3*, *PRPF6*, *PRPF8*, and *PRPF31*[28,30]. The progression of the disease is marked by night blindness and tunnel vision, and in later stages, complete blindness. However, no adverse phenotypes in non-retinal tissues are known [23,28].

There are two hypotheses that could explain how mutations in splicing factors result in RP [14]. First, because the pathology of the disease is restricted to the retina, RP may be the result of aberrant splicing of a transcript isoform that is specific to retinal tissue. Recently, transcriptome analysis of retinal tissue in a mouse model of RP revealed a large number of novel and/or aberrant transcripts [12]. This hypothesis does not rule out the second possibility: that these mutations in core components of the spliceosome increase the transcriptome-wide rate of splicing error and that retinal tissue is particularly sensitive to this increased rate of mis-splicing. Intermediates between these two extremes are also possible.

Here we test the hypothesis that a mutation in a splicing factor that causes RP leads to transcriptome-wide splicing defects in a non-retinal tissue. We used exon microarrays to profile transcript expression in whole blood samples obtained from RP-*PRPF8* individuals bearing the p.H2309R mutation in the splicing factor *PRPF8*[29] and paired sibling controls. For a large proportion of exons we found evidence of differential inclusion in mature transcripts of cases compared to controls. Differentially spliced exons were disproportionately associated with longer flanking introns and likely to be spliced through exon, rather than intron definition.

## Results

### Transcriptome-wide perturbation of splicing

We generated exon-level microarray expression data from four individuals with a mutation in *PRPF8*, a core component of the spliceosome, and sibling controls. Preprocessing and quality control steps were carried out as described in the Methods section. Following quality control steps and removal of probesets that were not expressed in at least 50% of the samples, a total of 103,268 core and 149,835 full probesets remained. To test for evidence of differential splicing we compared probeset inclusion (defined as the log of the ratio of probeset expression intensity to the expression intensity of the corresponding metaprobeset/transcript) between cases and controls. Comparisons were carried out separately for probesets in four overlapping categories, defined by whether the probeset mapped to an annotated intron or exon (referred to as the exonic and intronic categories, respectively) and by whether the probeset belonged to the core probesets or to the full probesets. The latter two categories are an aspect of the microarray design and reflect the confidence of the exon annotations on which the probesets were based. The core probesets correspond to high-confidence exon annotations, while the full probesets also target exons in computationally predicted transcripts. Intronic probesets were further partitioned based on splice type (major and minor).

**Figure 1:**
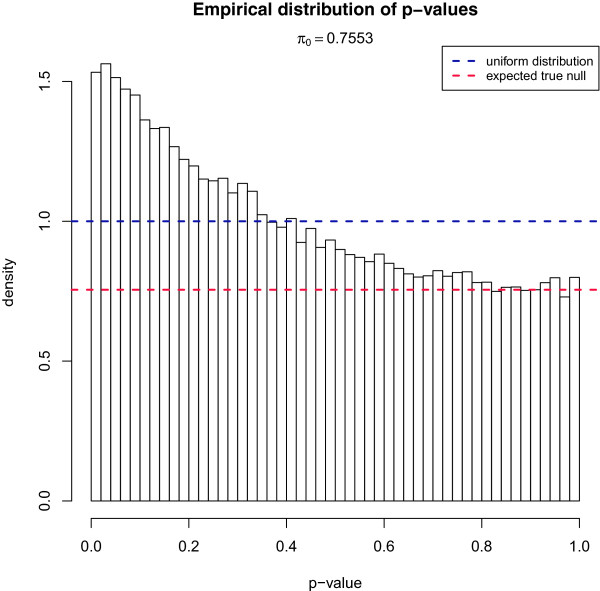
**Empirical distribution of *p *-values.** Each *p*-values was calculated using pairwise *t*-tests for a normalised probeset between cases and controls.

Probeset inclusion was compared between cases and sibling controls for each probeset that passed the detection and quality control filters described above. Instead of using limma we used standard *t*-tests so that we could estimate *π*_0_, the proportion of true null hypotheses. The limma approach has higher statistical power to detect differences at the individual probeset level, because it shares information across probesets but this violates the independence assumption when estimating *π*_0_. Consequently, the expected distribution of *p*-values is no longer uniform under the null hypothesis [31]. The histogram of *p*-values obtained from these comparisons (Figure 1) shows a large excess of small values compared to the uniform distribution, expected under a null hypothesis of no difference in splicing between cases and controls. Statistical methods exist to estimate *π*_0_ from a distribution of *p*-values. We used the qvalue package from BioConductor [32,33] for this purpose. For core probesets *π*_0_ was 0.76 while full probesets had a slightly higher value of 0.79. This suggests that for approximately one fifth of the probesets (roughly 24% for core and 21% for full probesets) the null hypothesis of no difference in inclusion between cases and controls does not hold. For exonic probesets *π*_0_ was 0.77 while intronic probesets had *π*_0_=0.81.

It is possible that this high proportion of affected probesets inferred using the qvalue package could be the result of a failure of the assumptions underlying the estimation of *π*_0_ and not a consequence of differences in splicing between the case and control groups. To investigate this possibility, we compared the estimates of *π*_0_ obtained from the true case-control groups to estimates obtained when sample labels were permuted exhaustively within sibling pairs (so that comparable paired *t*-tests could still be carried out). The estimate of *π*_0_ for the correctly labelled samples was lower than for any of the eight alternative configurations that can be obtained in this way (*p* = 0.125; Additional file 1: Figures S2 and S3). In a similar way, the value of *π*_0_ was lowest for all 35 possible case-control pairings, without regard to sibling relationships (*p*=0.03). The value of *π*_0_ was strongly anti-correlated with the number of properly paired sibling pairs (Pearson *r*=−0.84, *p*=0.008; Spearman *ρ*=−0.92, *p*=0.001), suggesting that the *PRPF8* mutation does have a transcriptome-wide effect on mRNA splicing in whole blood samples.

### Differential splicing primarily involves higher inclusion of exons in cases

**Figure 2:**
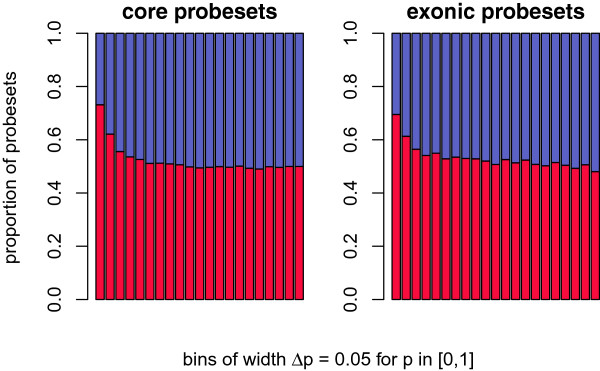
**Proportion of probesets indicating higher/lower inclusion in cases for binned *p *-value thresholds.** All bins have an equal width of *Δp*=0.05 for *p*-values in the range [0,1]. Red bars symbolise the proportion of probesets indicating higher inclusion in cases; similarly, blue bars refer to lower inclusion in cases. The absolute height of a bar of each colour represents the proportion of probesets with higher/lower inclusion in cases. The *t*-statistic was used to determine relative inclusion: *t*>0 - higher inclusion in cases; *t*<0 - lower inclusion in cases. Plot only shows core and exonic (full) probesets.

We used limma to compute *p*-values and *t*-statistics for differential inclusion of individual probesets because this approach has been shown to have higher statistical power than standard *t*-tests for small sample sizes [34]. The majority of probesets with significantly different inclusion between cases and controls had higher inclusion in cases (Figure 2; Additional file 2). Below a *p*-value threshold of 0.2, the enrichment for probesets with higher inclusion in cases was highly statistically significant (core probesets: *p*=4.8×10^−4^, full: *p*=1.3×10^−4^, exonic: 4.6×10^−10^; Additional file 1: Table S1–S3, Additional file 1: Figure S6). However, intronic probesets showed no significant excess of positive *t*-statistics among probesets with low *p*-values (*p*=0.24 at *p*-value threshold of 0.2; Additional file 1: Tables S4 and S5). This suggests that most of the differential splicing involves higher exon inclusion in cases. The affected probesets tended to be expressed at a lower level than the remainder of the probesets in the meta-probeset (gene) to which they mapped. Probesets with higher inclusion in cases had a mean expression intensity (across cases and controls) that was, on average, less than half the mean expression intensities of unaffected probesets (*p*=9.6×10^−112^). The difference was much smaller for probesets with lower inclusion in cases; these probesets had raw expression intensities that were on average 14% lower than the average of the unaffected probesets (*p*=0.004). Thus, the affected probesets are from low inclusion exons that are typically included at higher levels in the mutant samples compared to controls.

### Characterisation of differentially included exons

**Figure 3:**
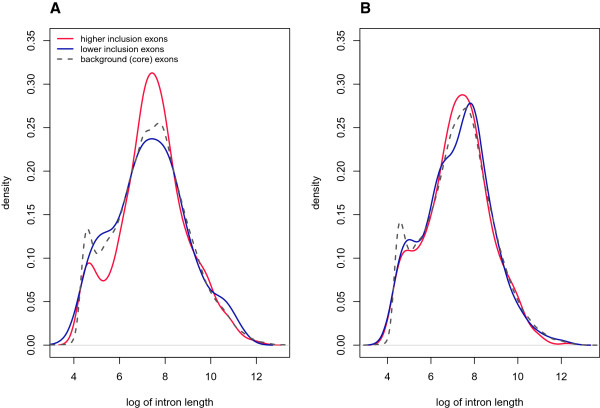
**Distributions of intron length.** Density plot of intron length **(A)** upstream and **(B)** downstream of differentially included exons in cases relative to controls. A similar plot for background exons (core exons) is provided for reference.

The introns upstream of exons containing probesets with evidence of differential inclusion between cases and controls were significantly longer than average (Table 1). Short introns (<250 bp) are thought to be excised primarily through *intron definition*, and longer introns through *exon definition*[35-37]. These two classes of introns can be seen on the density plot of intron lengths (Figure 2). The density plot of introns bordering differentially included exons had a diminished peak at short lengths (Figure 3), suggesting that aberrant splicing in cases does not affect splicing via intron definition. Instead, longer introns were particularly abundant upstream of preferentially included exons (Figure 3). Thus, differential splicing between cases and controls appears to primarily affect the exon definition pathway.

**Table 1:** Length of introns flanking differentially included exons

	**Upstream intron length (5’)**			**Downstream intron length (3’)**		
	**Median**	***p*-value**	***q*-value**	**Median**	***p*-value**	***q*-value**
**Up**	1966	0.002	0.0064	1662	0.84	0.84
**Down**	2053.5	0.05	0.1	1843.5	0.14	0.18
**All**	1625	-	-	1625	-	-

Comparison of median of intron length for exons with higher (up) and lower (down) inclusion in cases relative to controls.

Exon definition occurs for short to moderate length (<500 bp) exons since the components of the spliceosome need to associate across them [38]. Long exons tend to have additional splicing enhancer motifs, perhaps to aid the binding of spliceosome components since they cannot associate across the length of the exon [38]. We found that exons containing probesets with evidence of higher inclusion in cases than in controls were significantly shorter than average (Table 2). We also found that exons with lower inclusion in cases were somewhat longer than other exons (Table 2). Exons that showed evidence of higher inclusion in cases relative to controls were depleted of long exons (>500 bp), suggesting that the majority of these exons are spliced via exon definition [39].

**Table 2:** Length of differentially included exons

	**Mean**	**Median**	***p*-value**	***q*-value**
**Up**	368.88	127	7.52×10^−18^	1.50×10^−17^
**Down**	562.81	166	2.44×10^−3^	2.44×10^−3^
**All**	436.5	153	-	-

Comparison of mean and median lengths for exons with higher (up) and lower (down) inclusion in cases relative to controls.

We used MaxEntScan[40] to calculate splice site scores at intron-exon junctions and compared these between affected and unaffected exons. For exons with higher inclusion in cases, the median score of 5’ splice sites (5SS) was significantly higher than background (Tables 3 and 4) but the 3’ splice sites (3SS) showed no difference in score. In contrast, for exons with lower inclusion in cases, neither the 5SS nor 3SS showed differences in scores (Tables 3 and 4). The distributions of splice site scores are shown in Additional file 1: Figure S4.

**Table 3:** Splice site strength of differentially included exons

	**Splice donors (5SS)**				**Splice acceptors (3SS)**			
	**Mean**	**Median**	***p*-value**	***q*-value**	**Mean**	**Median**	***p*-value**	***q*-value**
**Up**	8.33	8.76	8.86×10^−3^	3.54×10^−2^	8.43	8.68	0.58	0.75
**Down**	8.12	8.68	0.75	0.75	8.19	8.40	0.10	0.19
**All**	8.08	8.68	-	-	8.30	8.69	-	-

Mean and median splice site strength computed using MaxEntScan[40].

**Table 4:** Combined splice site strength (5SS and 3SS) of differentially included exons

	**Sum of 5SS and 3SS scores**			
	**Mean**	**Median**	***p*-value**	***q*-value**
**Up**	16.76	17.12	0.04	0.08
**Down**	16.31	16.77	0.11	0.11
**All**	16.38	16.99	-	-

Similar to Table 3 but using combined splice site strength.

**Figure 4:**
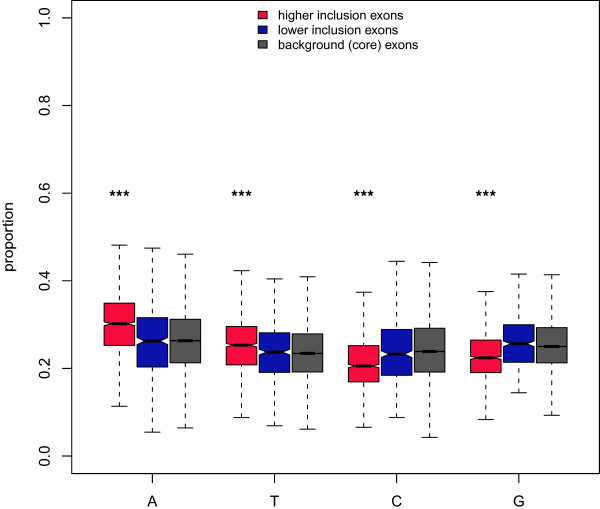
**Nucleotide content of differentially included exons.** The proportion of each nucleotide in differentially included exons.

We found that the exons with higher inclusion in cases had much higher proportions of A and T nucleotides than background (core) exons and, correspondingly, lower proportions of G and C nucleotides (Figure 4). Exons with lower inclusion in cases showed no significant difference from background. We also obtained a list of 238 candidate hexameric exonic splice enhancers (ESEs) [41]. For each ESE, we counted the number of times it occurred in each exon category and normalised this by the sum of exon lengths in that category. Unsurprisingly, considering that ESEs are A-rich (nearly 50% of bases of the 238 ESEs were A), we found that highly included exons did indeed show evidence of ESE-enrichment (mean ESE prevalence of 4.14×10^−4^ against 3.70×10^−4^ for core exons, *p*=0.02). We did not observe any ESE enrichment in exons with lower inclusion in cases (mean ESE prevalence of 3.76×10^−4^, *p*=0.72). The enrichment of ESEs in the exons with increased inclusion in cases does not explain the A+T richness because the excess of A and T nucleotides persisted even when all instances of the 238 ESEs were removed. Indeed, the excess of ESEs may be a consequence of the A-richness of the affected exons.

Splicing can take place co-transcriptionally (while the polymerase is still associated with the DNA template) or post-transcriptionally [42-48]. Exons belonging to longer genes or located far from the 3’ ends of genes are more likely than other exons to be spliced co-transcriptionally [49]. To investigate whether the mutation may have a greater impact on co- or post-transcriptional splicing we compared gene length and distance from 3’ gene ends between affected exons (differentially included between cases and controls) and unaffected exons. We found that the mean gene length as well as the distance from the 3’ end of the gene were both significantly greater for affected exons (Table 5). This suggests that the mutation has a larger impact on co-transcriptional splicing; the effect may even be restricted to splicing that occurs co-transcriptionally.

**Table 5:** Indicators of co-transcriptional splicing

	**Gene length**				**Distance from 3’ end**			
	**Mean**	**Median**	***p*-value**	***q*-value**	**Mean**	**Median**	***p*-value**	***q*-value**
**Up**	100,954	53,464	2.6×10^−7^	5.2×10^−7^	51,939	23,542	7.3×10^−10^	1.4×10^−9^
**Down**	103,781	53,741	3.9×10^−5^	3.9×10^−5^	58,279	21,426	5.1×10^−5^	5.1×10^−5^
**All**	91,811	42,729	-	-	46,338	14,514	-	-

Gene length and distance from the 3’ end of genes for exons differentially included.

## Discussion

*Cis*-acting factors have been shown to be important regulators of alternative splicing and several previous studies have reported *cis*-acting genetic variants that affect mRNA splicing [16,18-21,50-52]. Such *cis*-acting splicing quantitative trait loci (sQTLs) typically affect the splicing of a single gene. *Trans*-acting variants, however, can have widespread impact and when they affect ubiquitous components of the transcript processing machinery the effects may be transcriptome-wide. To date, there have been fewer studies on *trans*-sQTLs than on *cis*-acting variants. Given that almost all human genes are spliced and a large majority of multi-exon genes are alternatively spliced, often in a tissue-specific manner [53], *cis*- and *trans*-acting factors that cause splicing errors or affect the regulation of alternative splicing have the potential to have a substantial impact on the transcriptome. Indeed, a large proportion of human genetic diseases are likely to result from mutations that affect splicing [25,53-55].

In this study, we applied statistical analyses that interrogated exon inclusion events across the human transcriptome. We estimated the transcriptome-wide effect of the *PRPF8* mutation by using the *π*_0_ statistic, which in the context of multiple hypothesis testing, is an estimate of the proportion of tests that conform to the null hypothesis. This approach has previously been applied in transcriptome-wide studies of population differential gene expression between human populations [56] and, more recently, in the study of *cis*- and *trans*-expression quantitative loci [22]. The observed value of *π*_0_ was the lowest among all permutations of the case-control labels. Nevertheless, we acknowledge that, given the relatively small sample size in this study, this finding requires independent validation particularly given the often noisy nature of microarray data.

Our results provide evidence that a non-synonymous mutation in the splicing factor *PRPF8* that leads to retinitis pigmentosa affects the inclusion of approximately 20*%* of the exons in genes expressed in whole blood samples. Affected exons were most often included at a higher level in the transcripts of cases than of controls. Averaging across all samples (cases and controls), these exons had lower inclusion levels than unaffected exons, suggesting that they are absent from some of the transcripts of the corresponding gene. The fact that the mutated form of *PRPF8* was associated with higher inclusion of exons that appear to be skipped in some transcripts was an unexpected result. It suggests that the mutation may affect alternative splicing (e.g. tissue-specific regulation of alternative splice isoforms) rather than giving rise to a high rate of splicing errors involving skipping of constitutive exons or intron retention. Exon skipping is the most common type of alternative splicing event [57]. Because the mutation is associated with higher inclusion levels of exons that are expressed at relatively low levels, we propose that the *PRPF8* mutation may reduce the likelihood of exon skipping. Under this model the adverse phenotype could result from failure of a regulated exon skipping event which is required in retina. The exon microarray platform we used also includes some probesets that map to intronic regions. Although the number of expressed probesets mapping to introns was far lower than for exons, we found some evidence of differential intron retention between cases and controls. However, the proportion of affected probesets was lower for intronic compared to exonic probesets and the overall effect was approximately equally divided between increased and decreased intron inclusion.

The introns upstream of exons with higher inclusion in cases were significantly longer than average. In fact, short introns were almost entirely absent upstream of affected exons (Figure 3). Short introns (<250 bp) are more efficiently excised through the assembly of spliceosomal components across the intron before pairing up (intron definition) and they generally have weaker splice sites [58]. Long introns rely on spliceosome components first assembling across exons before juxtaposing across the target introns.

One of the most striking features of probesets with significantly different inclusion in cases compared to controls was that they were found predominantly on shorter exons. The majority of affected exons had increased inclusion in cases and we found that shorter exon length applied only to these exons. In fact, probesets with lower inclusion in cases compared to controls mapped to exons that were slightly longer than the background unaffected exons. Exon defined splicing is most efficient when exon length is between 50 and 500 bp [35,39,59]. Splicing via intron definition requires short introns (<250 bp) and, as discussed above, these were depleted among introns flanking affected exons, particularly for the exons with higher inclusion in cases. Consequently, we propose that the *PRPF8* mutation affects splicing via exon definition, but may have no effect on splicing via intron definition. We also observed that exons with higher inclusion in cases tended to have stronger 5’ splice site (5SS). Interestingly, *PRPF8* is known to interact directly with the 5SS [2] (and references therein). It contacts the 5SS dinucleotide at residues QACLK (positions 1894–1898) as part of the U5 snRNP (a constituent of the U5 ·U4/U6 tri-snRNP) [60-63]. However, the RP mutation is a histidine to arginine change at residue 2309 [29] suggesting that the mutation does not directly affect contact with the 5SS and may, instead, affect interaction with another protein or snRNA.

On average, exons that were differentially included between cases and controls were much further from 3’ gene ends and belonged to significantly longer genes than unaffected exons. Both distance from the 3’ end and gene length are associated with the efficiency of co-transcriptional splicing [49]. The majority of affected exons had higher inclusion in cases and exons with higher inclusion in cases (but not exons with lower inclusion) had significantly greater proportions of A and T nucleotides than unaffected exons. A-rich tracts have been observed to lead to pol II pausing [64,65], potentially increasing the time available for mRNA splicing to occur prior to the completion of transcription. Taking these observations together we propose that the *PRPF8* mutation may primarily or, at least, disproportionately affect splicing that takes place co-transcriptionally.

Two previous studies of the effects of mutations in three splicing factors (*PRPF3*, *PRPF8* and *PRPF31*) linked to retinitis pigmentosa on mRNA splicing in lymphoblast cells reported evidence of retained introns [11,14]. Both of these studies investigated splicing for a small number of introns. Only one intron showed evidence of retention associated with the mutation in *PRPF8*[14]. This was a U12-type intron (the third intron of *STK11*). However, the corresponding probesets did not show evidence of expression above background, thus we did not find evidence to support retention of this intron in our dataset. Furthermore, we did not find any evidence of retention of U12-type introns in cases (Additional file 1: Figure S5).

We observed that exons with specific structural characteristics were more likely to be differentially-included: short exons flanked by long introns, high A+T-content, located towards the 5’ end of the gene. It is possible that these exons are more prone to microarray hybridisation noise. For example, it can be more difficult to design microarray probesets targeting short exons (short sequences limit the choices of array probes), resulting in lower hybridisation affinity. Indeed, we observed higher variability of expression signals in affected exons compared to unaffected exons even when restricting to controls alone (data not shown). Nevertheless, given that sample preparation and processing was carried out independent of treatment labels, technical noise should not be biased towards increased exon inclusion in cases. Our results suggest that a mutation in *PRPF8*, implicated in RP, has a subtle effect on the inclusion of a large number of human exons. However, further investigation using newer sequencing based technologies (RNA-Seq) and an independent set of samples will enable the impact of the mutation on splicing error to be confirmed and dissected in greater detail.

## Conclusion

Overall, our results support the hypothesis that a mutation in the splicing factor *PRPF8* that leads to retinitis pigmentosa, has a widespread impact on mRNA splicing across the transcriptome. Given that splicing of such a large proportion of exons is effected by the mutation in blood, it is surprising that the phenotype associated with this mutation is restricted to the retina. However, because the differentially included probesets were from exons with low inclusion overall and had higher inclusion in cases compared to controls, we propose that the mutation does not lead to an increase in the rate of mis-splicing of constitutive exons. Instead, the mutation may influence the inclusion of alternatively spliced exons. Consequently, we suggest that the disease phenotype in retinal tissue could result from failure to produce one or several retina-specific isoforms that require exon skipping.

## Methods

### Exon array sample preparation and data generation

This project was approved by the University of Cape Town Research Ethics Committee (REC REF: 180/2009), which is in compliance with the guidelines of the declaration of Helsinki. Written informed consent for participation in the study was obtained from participants.

Blood from five individuals carrying the autosomal dominant RP mutation p.H2309R (referred to as *cases*) together with that from unaffected sibling controls was collected and preparation coordinated to ensure equal sample incubation times. Three blood samples per subject were collected into PAXgene™ tubes (PreAnalytiX), according to the manufacturer’s instructions, and incubated at room temperature. RNA extractions were performed 16 hours (A sample), 20 hours (B sample) and 39 hours (C sample) after collection. Total RNA was extracted using the PAXgene™ Blood RNA kit, following the manufacturer’s recommended protocol. The RNA samples were not heat denatured following elution, but immediately stored at -80°C.

The A and B RNA isolations of each sample were pooled, and the Affymetrix GeneChip®; Blood RNA Concentration Kit used to concentrate the RNA. Quality control checks to determine RNA concentration and integrity were performed for each sample, using the Nanodrop (Thermo Scientific) and Agilent 2100 Bioanalyser (Agilent Technologies), respectively. The RNA integrity number (RIN) quality threshold used was 7. It was determined that one of the cases showed poor integrity of A/B and C RNA samples leading to its exclusion together with its sibling-pair. RNA yields of between 2.22 and 5.16 *μ*g were obtained for the remaining samples, which were of satisfactory integrity to proceed with microarray analysis. The 260/280 ratios for the samples ranged from 1.97 to 2.06. All samples exceeded the RNA integrity number (RIN) quality threshold ≥7, as samples ranged from RIN 8.3–9.10.

Ribosomal RNA reduction was performed on the eight remaining samples using the RiboMinus™ Transcriptome Isolation Kit (Human/Mouse) (Invitrogen), in accordance with a modified Affymetrix protocol. The RNA was then processed with the Whole Transcript (WT) Sense Target Labelling assay. The labelled samples were hybridised to Affymetrix GeneChip®; Human Exon Array 1.0 ST chips according to the prescribed protocol. A total of 5.5 *μ*g of single stranded cDNA (generated from cRNA) was hybridised to the arrays. Following hybridization, the arrays were scanned in the Affymetrix GeneChip®; Scanner 3000 7G.

Cases were labelled *T*_1_, *T*_2_, *T*_3_, and *T*_4_ and corresponding sibling controls *C*_1_, *C*_2_, *C*_3_, and *C*_4_.

### Array quantification and intensity normalisation

Raw exon array intensities were summarised into probesets and metaprobesets at the core and full probeset/metaprobeset level using Affymetrix Power Tools (APT) by the robust multi-chip averaging (RMA) algorithm [66]. Quality control of array data was carried out according to a previously described procedure [67]. We applied hierarchical clustering and principal components analysis to the raw intensities to identify possible outliers (Additional file 1: Figure S1). APT was also used to determine probesets and metaprobesets that were detectable above the background (DABG) [68]. We excluded from further analysis all probesets that were detectable above background in fewer than half of the samples. We also used the annotation files provided by Affymetrix to exclude all probesets with probes likely to cross-hybridise. A similar procedure was applied to metaprobesets: only those having at least half of the associated probesets expressed above background in all samples were retained [67]. Finally, we normalised each probeset intensity by subtracting its value from its parent metaprobeset intensity since these values lie on a logarithmic scale [69]. The number of probesets remaining after these filtering steps were 103,268 and 149,835 for the core and full sets, respectively.

All analyses were performed on the hg19 build of the human genome. Exon boundaries and splice sites were defined based on build 66 of the Ensembl database of gene models [70]. Exonic and intronic probesets were isolated using the R tool xmapcore, using the database based on build 66 of the Ensembl gene model [71]. U12-type introns were obtained from the U12DB [72] with coordinates modified to hg19 using liftOver[73].

### Testing for differential splicing

For each probeset, log-transformed expression intensities of probesets were compared between cases and controls. The log-transformed expression intensities were normalized by subtracting the log expression intensities of corresponding metaprobesets from that of probesets. We compared the expression intensity for each probeset using paired tests first using Welch *t*-tests from the standard R library then using moderated paired *t*-tests implemented in the limma[34] package. Correction for multiple testing was based on the *q*-value method as implemented in the R package qvalue[32]. The qvalue package was also used to estimate *π*_0_, the expected proportion of tests consistent with the null hypothesis.

### Characterisation of differentially spliced exons

To avoid the potential for bias resulting from exons to which multiple probesets mapped or individual probesets that mapped to multiple overlapping exons, we constructed a one-to-one mapping of probesets to exons by randomly sampling at most one exon for each probeset and one probeset for each exon. We compared the characteristics of exons for which the corresponding probeset was differentially included between cases and controls using a significance threshold of 0.01. Exons with significantly higher and lower inclusion levels in cases relative to controls were considered separately and, in each category, eight features of the exon (5’ and 3’ splice site scores, length of up and downstream introns, exon length, nucleotide content, distance from the 3’ end and associated gene length) were compared between the significantly included/excluded exons and the remainder of the exons tested. For each feature, we compared groups using a non-parametric test (Wilcoxon rank-sum test), followed by correction for multiple testing either using the Benjamini-Hochberg method [74] or the *q*-value method [32].

## Availability of supporting data

Affymetrix exon array CEL files are available on GEO website under accession GSE43134 (http://www.ncbi.nlm.nih.gov/geo/query/acc.cgi?acc=GSE43134). This manuscript is accompanied by supplementary information.

## Abbreviations

PRPF8: pre-mRNA processing factor 8.

## Competing interests

The authors declare no competing financial interests.

## Authors’ contributions

CS conceived the project. RR and CS supervised the project. LR performed lab experiments. PK performed the analysis. PK and CS drafted the manuscript. All authors read and approved the final manuscript.

## Supplementary Material

Additional file 1 **Supplementary information.** Contains supplementary results mentioned in the text. Click here for file

Additional file 2 **Supplementary data.** Contains the list of nominally deregulated exons, their normalised expression estimates and key statistics. Click here for file
