# Supplementary material for: A mutation in a splicing factor that causes retinitis pigmentosa has a transcriptome-wide effect on mRNA splicing
Source: BMC Res Notes. 2014 Jun 27;7:401. doi: 10.1186/1756-0500-7-401 (PMC4084799; doi:10.1186/1756-0500-7-401)
Supplement: Additional file 1 — Supplementary information. Contains supplementary results mentioned in the text. [file 1756-0500-7-401-S1.pdf]

Supplementary Information
for
A mutation in a splicing factor that causes retinitis pigmentosa has a transcriptome-wide effect on mRNA splicing

Paul K. Korir, Lisa Roberts, Raj Ramesar, Cathal Seoighe

Contents

1 Quality Assessment **2**

2 Sample Permutations **3**
2.1 Core Probesets 3
2.2 Full Probesets 4

3 Additional Analyses **5**
3.1 Splice Site Scores 5
3.2 Constitutive versus Alternative Splicing Analyses 5
3.3 U12-type Introns 6

4 Number of Differentially Included Probesets **7**
4.1 Differential Inclusion by Probeset Class 7
4.2 Core Probesets 8
4.3 Full Probesets 9
4.4 Exonic Probesets 10
4.5 Intronic Probesets 11
4.6 U12-type Intronic Probesets 12

5 Transcript Cluster Identifiers (Metaprobesets) Associated with *PRPF8* **12**

6 Associated Genes **13**
6.1 Higher Inclusion in Cases 13
6.2 Lower Inclusion in Cases 17

7 Associated Exons **19**
7.1 Higher Inclusion in Cases 19
7.2 Lower Inclusion in Cases 24

1 Quality Assessment

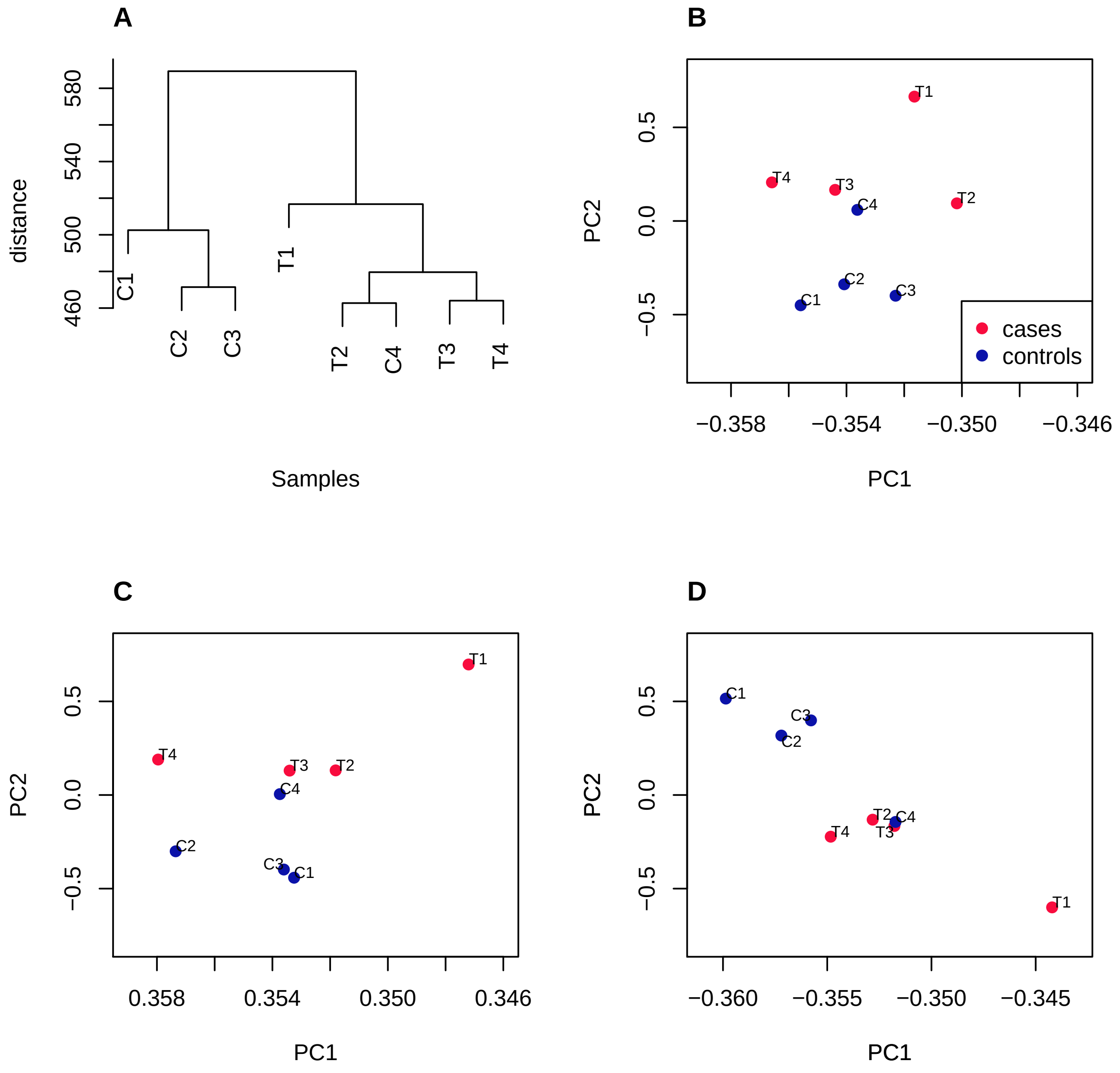

Figure S1: **Quality assessment of exon array data. A.** Hierarchical cluster of samples. T_i are *cases*, C_i are *controls*. **B.** Plot of first two principal components (PCs) for full probesets. **C.,D.** First two PCs for exonic and intronic probesets, respectively.

2 Sample Permutations

2.1 Core Probesets

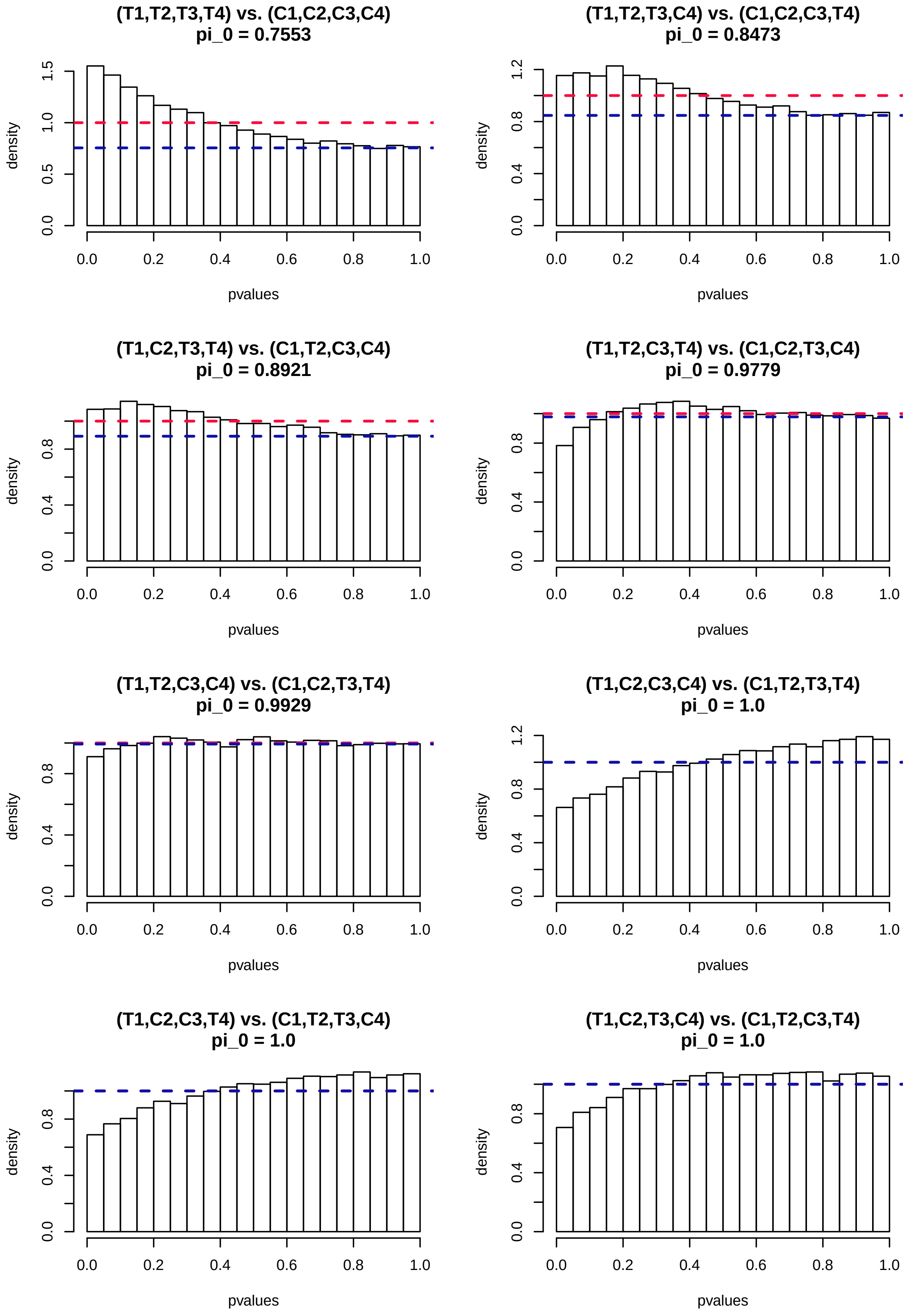

Figure S2: **Sample permutations for core probesets.** Plots representing the complete set (eight) of pairwise swaps between cases (T_i) and controls (C_i). Only core probesets are considered here. Plots are ordered from the top by the value of π_0. Dashed red line represents a uniform distribution; dashed blue lines represent the value of π_0.

2.2 Full Probesets

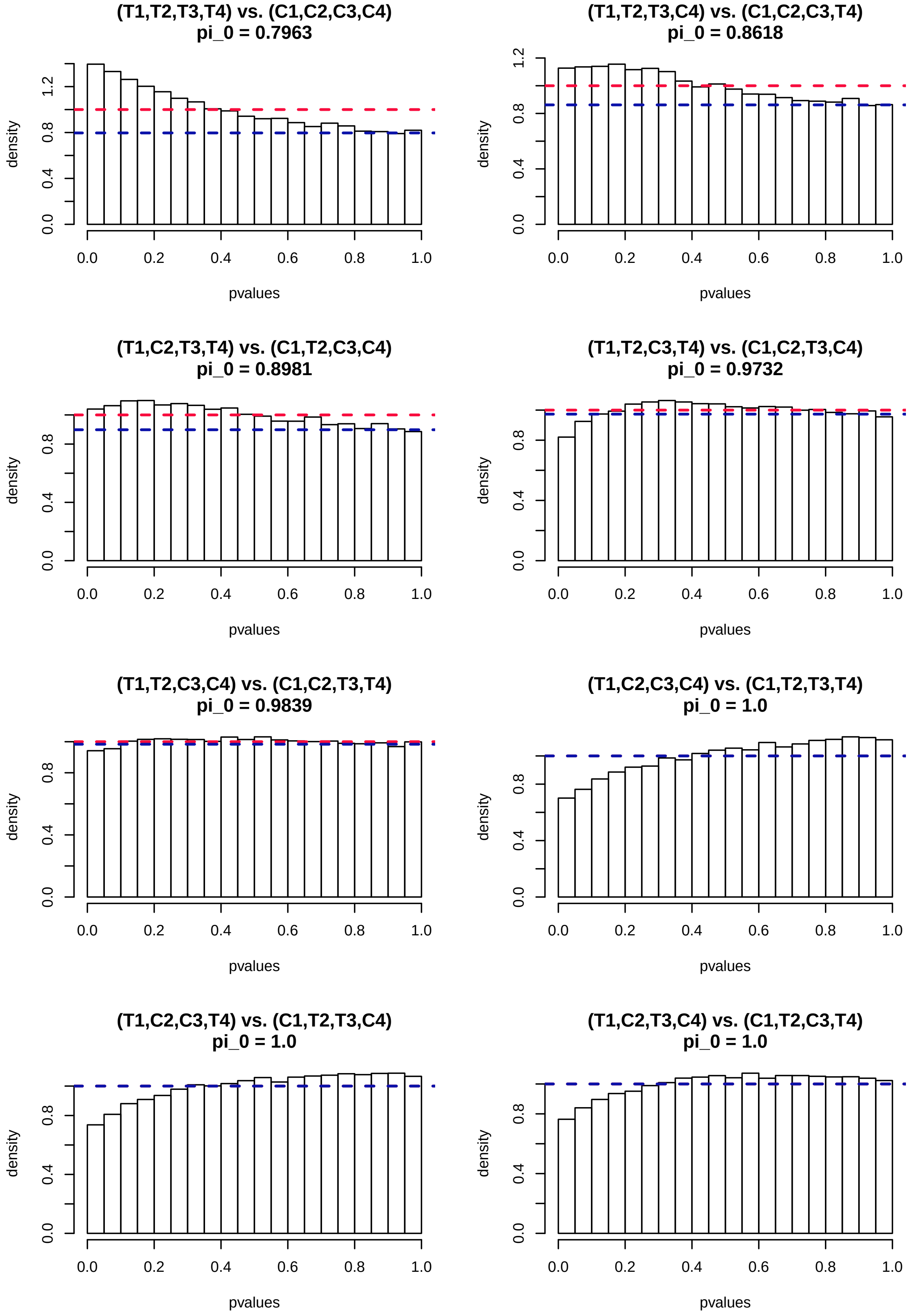

Figure S3: **Sample permutations for full probesets.** Plots representing the complete set (eight) of pairwise swaps between cases (T_i) and controls (C_i). Plots are ordered from the top by the value of π_0. Dashed red line represents a uniform distribution; dashed blue lines represent the value of π_0.

3 Additional Analyses

3.1 Splice Site Scores

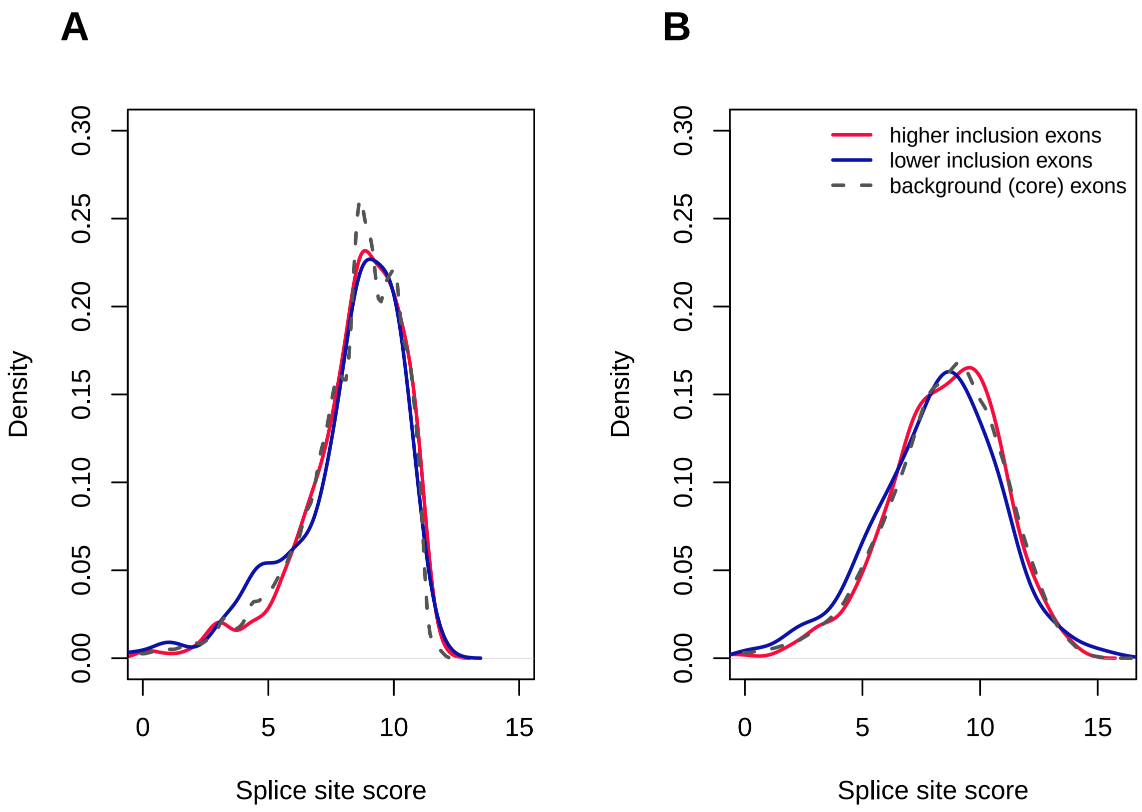

Figure S4: **Distribution of splice site scores.** Density plot of splice site scores of **A.** 5' splice sites and **B.** 3' splice sites. The dashed line indicates a similar plot for background exons (core exons).

3.2 Constitutive versus Alternative Splicing Analyses

We reasoned that exons differentially included would also be enriched for alternatively spliced exons. However, we found no evidence of splicing bias for exons prone to alternative splicing. We tested this by constructing reference lists for constitutive and alternative exons in lymphoblastoid (similar to the whole blood samples we used) tissue [1] using the following procedure.

First, we aligned RNA-Seq data for 53 YRI samples to the hg19 build of the genome and isolated all junction spanning reads which numbered about 62 million reads. We then constructed a reference set of exons from the Ensembl gene model. Exons were defined at the transcript level by associating each transcript ID with the exon number (e.g. `ENST*:1` for the first exon in `ENST*`). This method produced a redundant list because many exons overlap. Overlapping exons were eliminated using a randomisation procedure: from every set of exons that shared at least one terminal coordinate (start/stop) one exon was chosen at random.

For each internal exon we counted the number of reads that mapped to the upstream ($C_1 : A$) and downstream ($A : C_2$) junction as well as those that mapped only to flanking exons (skipping)

$(C_1 : C_2)$ [2]. We computed the percent-/proportion-spliced-in (PSI or Ψ) using the expression

$$\Psi = \frac{\text{avg}(C_1 : A, A : C_2)}{\text{avg}(C_1 : A, A : C_2) + C_1 : C_2}.$$

An exon was considered constitutive if $\Psi - 2 \cdot \text{SE}(\Psi) > 0.5$ and alternative if $\Psi + 2 \cdot \text{SE}(\Psi) < 0.5$. In this way we identified 19,685 constitutive and 3,078 alternative exons. From this list of constitutive and alternative exons we tallied those present in higher/lower inclusion in cases and compared them to similar counts in the background set of exons using Fisher's exact test. For exons included at higher levels in cases the odds ratio was 1.57 ($p = 0.45$) while those included at lower levels had $OR = 3.59$ ($p = 0.27$) suggesting no splicing-specific influence.

3.3 U12-type Introns

We did not find any distinction in probeset intensities based on intron type. U2-type introns form the majority of introns and Fig. S6D adequately represents them. U12-type introns had a less even distribution (Supplementary Fig.S5) likely due to few probesets associated with them. No significant bias in inclusion levels was observed. We also compared U2-type and U12-type introns to each other based on their t-statistics. We found no significant difference in the t-statistics ($p = 0.6268$, 95% CI of difference of means: $[-0.27, 0.17]$) suggesting similar distributions for both.

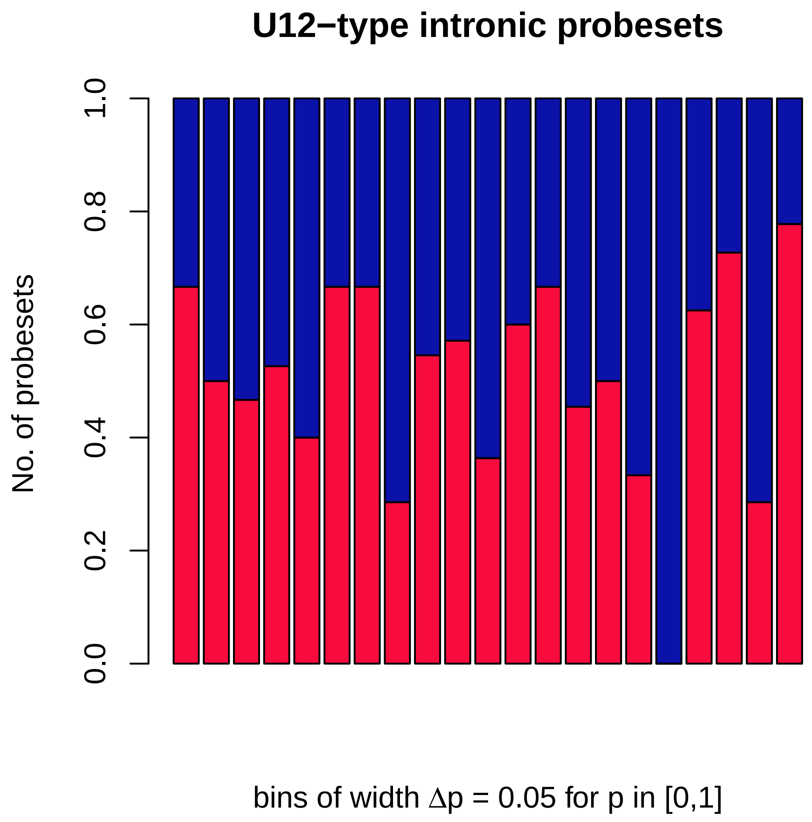

Figure S5: **Proportion of probesets indicating higher/lower inclusion in cases relative to controls for binned p-value thresholds for U12-type introns.** Each column represents all probesets (exons) for that bin. Red represent probesets included at higher levels in cases; blue are those included at lower levels in cases.

4 Number of Differentially Included Probesets

4.1 Differential Inclusion by Probeset Class

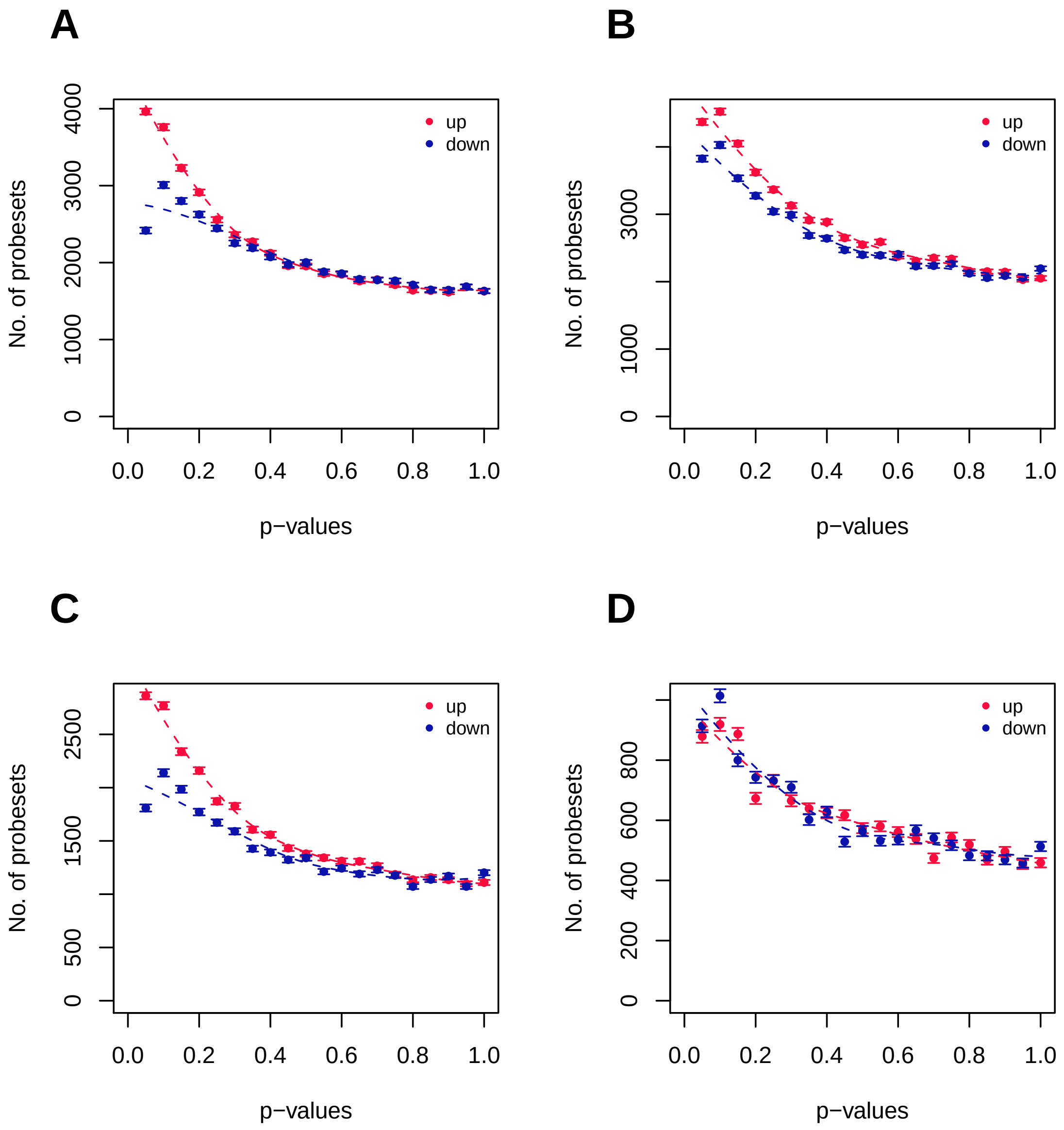

Figure S6: **Illustration of differential inclusion for different classes of probesets. A.** Core, **B.** full, **C.** exonic, and **D.** intronic probesets. Each point represents the number of probesets presenting higher ('up')/lower ('down') inclusion in cases relative to controls within $\Delta p = 0.05$ p-value bins.

4.2 Core Probesets

p **threshold**	**down**	**up**	**equal**	p**-value**	**FDR**
0.01	473	1287	0	7.64×10^{-87}	1.61×10^{-85}
0.05	2417	3963	0	3.61×10^{-84}	3.79×10^{-83}
0.1	3008	3760	0	6.36×10^{-20}	4.45×10^{-19}
0.15	2801	3230	0	3.52×10^{-8}	1.85×10^{-7}
0.2	2625	2913	0	1.2×10^{-4}	4.8×10^{-4}
0.25	2446	2557	0	0.12	0.36
0.3	2253	2362	0	0.11	0.36
0.35	2190	2271	0	0.23	0.58
0.4	2073	2122	0	0.46	0.81
0.45	1975	1959	0	0.81	0.99
0.5	2003	1956	0	0.46	0.81
0.55	1881	1856	0	0.69	0.99
0.6	1857	1848	0	0.90	0.99
0.65	1784	1761	0	0.71	0.99
0.7	1774	1779	0	0.95	0.99
0.75	1764	1713	0	0.40	0.81
0.8	1710	1642	0	0.25	0.58
0.85	1646	1637	0	0.89	0.99
0.9	1643	1617	0	0.66	0.99
0.95	1688	1685	0	0.97	0.99
1	1631	1629	207	0.99	0.99

Table S1: **Number of core probesets in each bin of width $\Delta p = 0.05$.** Data used to produce the plots in Fig. 2 and Fig. S6.

4.3 Full Probesets

p threshold	down	up	equal	p-value	FDR
0.01	1147	1400	0	5.82×10^{-7}	3.054×10^{-6}
0.05	3825	4370	0	1.84×10^{-9}	3.86×10^{-8}
0.1	4028	4524	0	8.60×10^{-8}	6.02×10^{-7}
0.15	3534	4048	0	3.78×10^{-9}	3.40×10^{-8}
0.2	3275	3622	0	3.09×10^{-5}	0.00013
0.25	3040	3366	0	4.88×10^{-5}	0.00017
0.3	2991	3129	0	0.080	0.1291
0.35	2685	2913	0	0.0024	0.0063
0.4	2642	2887	0	0.0010	0.0031
0.45	2470	2650	0	0.012	0.026
0.5	2399	2548	0	0.035	0.062
0.55	2391	2590	0	0.0050	0.012
0.6	2408	2378	0	0.68	0.68
0.65	2230	2306	0	0.27	0.35
0.7	2237	2352	0	0.093	0.14
0.75	2263	2337	0	0.28	0.35
0.8	2123	2155	0	0.64	0.68
0.85	2058	2150	0	0.16	0.23
0.9	2089	2143	0	0.42	0.48
0.95	2059	2032	0	0.68	0.68
1	2194	2052	25057	0.030	0.058

Table S2: **Number of full probesets in each bin of width** $\Delta p = 0.05$

4.4 Exonic Probesets

p **threshold**	**down**	**up**	**equal**	p**-value**	**FDR**
0.01	347	790	0	2.95×10^{-40}	4.59×10^{-40}
0.05	1809	2862	0	6.61×10^{-54}	2.059×10^{-53}
0.1	2139	2769	0	2.46×10^{-19}	2.55×10^{-19}
0.15	1985	2338	0	8.48×10^{-8}	5.29×10^{-8}
0.2	1771	2160	0	5.89×10^{-10}	4.59×10^{-10}
0.25	1672	1872	0	0.00083	0.00037
0.3	1590	1827	0	5.37×10^{-5}	2.79×10^{-5}
0.35	1427	1607	0	0.0012	0.00045
0.4	1392	1558	0	0.0024	0.00082
0.45	1323	1432	0	0.040	0.010
0.5	1340	1379	0	0.47	0.085
0.55	1212	1342	0	0.011	0.0033
0.6	1243	1311	0	0.19	0.038
0.65	1190	1308	0	0.019	0.0055
0.7	1229	1266	0	0.47	0.085
0.75	1176	1186	0	0.85	0.13
0.8	1072	1136	0	0.18	0.038
0.85	1138	1157	0	0.71	0.11
0.9	1170	1136	0	0.49	0.085
0.95	1072	1098	0	0.59	0.097
1	1202	1110	649	0.058	0.014

Table S3: **Number of exonic probesets in each bin of width** $\Delta p = 0.05$

4.5 Intronic Probesets

p **threshold**	**down**	**up**	**equal**	p**-value**	**FDR**
0.01	280	304	0	0.34	0.52
0.05	914	879	0	0.42	0.52
0.1	1014	919	0	0.033	0.17
0.15	800	887	0	0.036	0.17
0.2	743	673	0	0.067	0.24
0.25	731	732	0	1	0.85
0.3	710	665	0	0.24	0.52
0.35	602	639	0	0.31	0.52
0.4	628	624	0	0.93	0.83
0.45	529	617	0	0.010	0.17
0.5	564	574	0	0.79	0.78
0.55	532	580	0	0.16	0.40
0.6	536	561	0	0.47	0.52
0.65	567	538	0	0.40	0.52
0.7	541	474	0	0.038	0.17
0.75	517	543	0	0.44	0.52
0.8	483	519	0	0.27	0.52
0.85	482	468	0	0.67	0.71
0.9	469	496	0	0.40	0.52
0.95	458	453	0	0.89	0.83
1	513	459	4235	0.089	0.26

Table S4: **Number of intronic probesets in each bin of width** $\Delta p = 0.05$

4.6 U12-type Intronic Probesets

p threshold	down	up	equal	p-value	FDR
0.01	1	2	0	1	1
0.05	9	9	0	1	1
0.1	8	7	0	1	1
0.15	9	10	0	1	1
0.2	6	4	0	0.75	1
0.25	3	6	0	0.51	1
0.3	3	6	0	0.51	1
0.35	10	4	0	0.18	1
0.4	5	6	0	1	1
0.45	6	8	0	0.79	1
0.5	7	4	0	0.55	1
0.55	6	9	0	0.61	1
0.6	3	6	0	0.51	1
0.65	6	5	0	1	1
0.7	3	3	0	1	1
0.75	6	3	0	0.51	1
0.8	5	0	0	0.063	1
0.85	3	5	0	0.73	1
0.9	3	8	0	0.23	1
0.95	5	2	0	0.45	1
1	2	7	40	0.18	1

Table S5: **Number of U12-type intronic probesets in each bin of width** $\Delta p = 0.05$

5 Transcript Cluster Identifiers (Metaprobesets) Associated with *PRPF8*

PRPF8 is located on the reverse strand of chromosome 17 between base 1553923 and 1588154 (hg19 and EnsEMBL v.66). The following table shows corresponding metaprobesets.

ID	chr	strand	start	end
3705896	chr17	+	1577504	1577972
3705894	chr17	+	1574104	1574222
3705898	chr17	+	1581163	1581608
3740589	chr17	-	1574051	1574859
3740625	chr17	-	1583120	1583533
3740649	chr17	-	1587369	1587620

Table S6: **Metaprobesets along *PRPF8*.** The coordinates of transcript cluster IDs along the *PRPF8* gene.

Only 3740589 and 3740625 passed quality control filtering but were not differentially expressed between cases and controls.

6 Associated Genes

6.1 Higher Inclusion in Cases

ENSG00000000971 ENSG00000001036 ENSG00000001631 ENSG00000003147 ENSG00000003393
ENSG00000003509 ENSG00000004660 ENSG00000004809 ENSG00000004975 ENSG00000005249
ENSG00000005302 ENSG00000005471 ENSG00000005700 ENSG00000006432 ENSG00000006634
ENSG00000006659 ENSG00000006831 ENSG00000007264 ENSG00000007350 ENSG00000007516
ENSG00000007923 ENSG00000008083 ENSG00000008441 ENSG00000009307 ENSG00000009335
ENSG00000009954 ENSG00000010404 ENSG00000010704 ENSG00000011007 ENSG00000011028
ENSG00000011295 ENSG00000011566 ENSG00000012983 ENSG00000013306 ENSG00000013364
ENSG00000013725 ENSG00000014138 ENSG00000014216 ENSG00000017260 ENSG00000018510
ENSG00000023287 ENSG00000023318 ENSG00000023516 ENSG00000024048 ENSG00000028116
ENSG00000029363 ENSG00000033122 ENSG00000034677 ENSG00000034693 ENSG00000039068
ENSG00000040933 ENSG00000042832 ENSG00000042980 ENSG00000044115 ENSG00000047249
ENSG00000047365 ENSG00000047410 ENSG00000047597 ENSG00000047617 ENSG00000048392
ENSG00000048707 ENSG00000049246 ENSG00000049323 ENSG00000049541 ENSG00000049759
ENSG00000049860 ENSG00000049883 ENSG00000052841 ENSG00000053524 ENSG00000054219
ENSG00000054654 ENSG00000055917 ENSG00000055955 ENSG00000056586 ENSG00000058056
ENSG00000058272 ENSG00000059378 ENSG00000059588 ENSG00000060140 ENSG00000060339
ENSG00000061676 ENSG00000061987 ENSG00000062725 ENSG00000064042 ENSG00000064102
ENSG00000064313 ENSG00000064393 ENSG00000064703 ENSG00000064763 ENSG00000064961
ENSG00000065150 ENSG00000065243 ENSG00000065413 ENSG00000065485 ENSG00000065809
ENSG00000065882 ENSG00000066855 ENSG00000066923 ENSG00000067066 ENSG00000067113
ENSG00000067704 ENSG00000067798 ENSG00000067900 ENSG00000068878 ENSG00000068971
ENSG00000069493 ENSG00000069696 ENSG00000069943 ENSG00000070159 ENSG00000070182
ENSG00000070476 ENSG00000070961 ENSG00000071054 ENSG00000071537 ENSG00000072062
ENSG00000072364 ENSG00000072415 ENSG00000072756 ENSG00000072849 ENSG00000072952
ENSG00000073670 ENSG00000073803 ENSG00000074054 ENSG00000074276 ENSG00000074356
ENSG00000074582 ENSG00000075275 ENSG00000075340 ENSG00000075651 ENSG00000075785
ENSG00000075826 ENSG00000075884 ENSG00000075945 ENSG00000077097 ENSG00000077274
ENSG00000077522 ENSG00000077713 ENSG00000077721 ENSG00000078328 ENSG00000078618
ENSG00000078747 ENSG00000079263 ENSG00000079308 ENSG00000079805 ENSG00000079819
ENSG00000079841 ENSG00000080298 ENSG00000080345 ENSG00000080546 ENSG00000080823
ENSG00000081189 ENSG00000081923 ENSG00000082196 ENSG00000082258 ENSG00000082438
ENSG00000082898 ENSG00000083093 ENSG00000083168 ENSG00000083642 ENSG00000084093
ENSG00000084234 ENSG00000084754 ENSG00000084774 ENSG00000085491 ENSG00000085511
ENSG00000085563 ENSG00000085719 ENSG00000085733 ENSG00000086232 ENSG00000086758
ENSG00000086827 ENSG00000087245 ENSG00000087253 ENSG00000087274 ENSG00000087494
ENSG00000087586 ENSG00000088205 ENSG00000088356 ENSG00000088827 ENSG00000088832
ENSG00000088930 ENSG00000088970 ENSG00000089022 ENSG00000089048 ENSG00000089060
ENSG00000089091 ENSG00000089094 ENSG00000089169 ENSG00000089234 ENSG00000089505
ENSG00000089916 ENSG00000090238 ENSG00000090905 ENSG00000091039 ENSG00000091106
ENSG00000091129 ENSG00000091157 ENSG00000091409 ENSG00000091436 ENSG00000092094
ENSG00000092295 ENSG00000092330 ENSG00000092377 ENSG00000092758 ENSG00000092929
ENSG00000093167 ENSG00000094880 ENSG00000095059 ENSG00000095066 ENSG00000095321
ENSG00000096093 ENSG00000096401 ENSG00000099834 ENSG00000099910 ENSG00000099940
ENSG00000099991 ENSG00000100077 ENSG00000100151 ENSG00000100225 ENSG00000100281
ENSG00000100290 ENSG00000100294 ENSG00000100325 ENSG00000100330 ENSG00000100393
ENSG00000100401 ENSG00000100416 ENSG00000100592 ENSG00000100604 ENSG00000100612
ENSG00000100644 ENSG00000100650 ENSG00000100796 ENSG00000101017 ENSG00000101040
ENSG00000101146 ENSG00000101294 ENSG00000101307 ENSG00000101442 ENSG00000101751

ENSG00000102043 ENSG00000102098 ENSG00000102125 ENSG00000102174 ENSG00000102189
ENSG00000102543 ENSG00000102699 ENSG00000102753 ENSG00000102893 ENSG00000102897
ENSG00000102900 ENSG00000103257 ENSG00000103335 ENSG00000103342 ENSG00000103365
ENSG00000103522 ENSG00000103657 ENSG00000103876 ENSG00000103942 ENSG00000104154
ENSG00000104321 ENSG00000104341 ENSG00000104774 ENSG00000104812 ENSG00000104957
ENSG00000105370 ENSG00000105383 ENSG00000105393 ENSG00000105402 ENSG00000105497
ENSG00000105609 ENSG00000105699 ENSG00000105810 ENSG00000105939 ENSG00000105963
ENSG00000106012 ENSG00000106080 ENSG00000106327 ENSG00000106443 ENSG00000106546
ENSG00000106605 ENSG00000106688 ENSG00000106804 ENSG00000107201 ENSG00000107242
ENSG00000107262 ENSG00000107537 ENSG00000107581 ENSG00000107625 ENSG00000107672
ENSG00000107758 ENSG00000107862 ENSG00000107897 ENSG00000108094 ENSG00000108100
ENSG00000108278 ENSG00000108309 ENSG00000108344 ENSG00000108387 ENSG00000108389
ENSG00000108576 ENSG00000108592 ENSG00000108799 ENSG00000108819 ENSG00000109458
ENSG00000109685 ENSG00000110497 ENSG00000110628 ENSG00000110723 ENSG00000110799
ENSG00000110911 ENSG00000111142 ENSG00000111231 ENSG00000111291 ENSG00000111371
ENSG00000111554 ENSG00000111615 ENSG00000111644 ENSG00000111647 ENSG00000111666
ENSG00000111696 ENSG00000111783 ENSG00000111799 ENSG00000111845 ENSG00000111886
ENSG00000112038 ENSG00000112146 ENSG00000112186 ENSG00000112200 ENSG00000112249
ENSG00000112294 ENSG00000112494 ENSG00000112511 ENSG00000112799 ENSG00000112893
ENSG00000112983 ENSG00000112996 ENSG00000113108 ENSG00000113318 ENSG00000113327
ENSG00000113369 ENSG00000113407 ENSG00000113441 ENSG00000113580 ENSG00000113595
ENSG00000113645 ENSG00000113719 ENSG00000113946 ENSG00000113971 ENSG00000114030
ENSG00000114098 ENSG00000114127 ENSG00000114166 ENSG00000114209 ENSG00000114248
ENSG00000114279 ENSG00000114302 ENSG00000114439 ENSG00000114626 ENSG00000114739
ENSG00000114757 ENSG00000114770 ENSG00000114784 ENSG00000114861 ENSG00000114867
ENSG00000114978 ENSG00000115009 ENSG00000115053 ENSG00000115091 ENSG00000115109
ENSG00000115267 ENSG00000115271 ENSG00000115295 ENSG00000115464 ENSG00000115947
ENSG00000115966 ENSG00000116062 ENSG00000116117 ENSG00000116299 ENSG00000116539
ENSG00000116580 ENSG00000116586 ENSG00000116675 ENSG00000116679 ENSG00000116748
ENSG00000116957 ENSG00000117523 ENSG00000117614 ENSG00000117625 ENSG00000117697
ENSG00000117713 ENSG00000118004 ENSG00000118193 ENSG00000118420 ENSG00000118473
ENSG00000118707 ENSG00000118777 ENSG00000118873 ENSG00000118900 ENSG00000119041
ENSG00000119121 ENSG00000119139 ENSG00000119314 ENSG00000119318 ENSG00000119383
ENSG00000119487 ENSG00000119522 ENSG00000119685 ENSG00000119772 ENSG00000119782
ENSG00000119820 ENSG00000119950 ENSG00000120029 ENSG00000120253 ENSG00000120693
ENSG00000120756 ENSG00000120899 ENSG00000121486 ENSG00000121542 ENSG00000121848
ENSG00000121892 ENSG00000121895 ENSG00000122008 ENSG00000122025 ENSG00000122068
ENSG00000122223 ENSG00000122299 ENSG00000122484 ENSG00000122643 ENSG00000122687
ENSG00000122756 ENSG00000122783 ENSG00000122958 ENSG00000123104 ENSG00000123200
ENSG00000123213 ENSG00000123240 ENSG00000123496 ENSG00000123505 ENSG00000123607
ENSG00000123609 ENSG00000123636 ENSG00000123815 ENSG00000123836 ENSG00000123892
ENSG00000124193 ENSG00000124207 ENSG00000124214 ENSG00000124215 ENSG00000124356
ENSG00000124571 ENSG00000124574 ENSG00000124596 ENSG00000124767 ENSG00000124782
ENSG00000124788 ENSG00000125107 ENSG00000125257 ENSG00000125375 ENSG00000125676
ENSG00000125695 ENSG00000125863 ENSG00000125952 ENSG00000126070 ENSG00000126091
ENSG00000126231 ENSG00000126777 ENSG00000126870 ENSG00000127328 ENSG00000127481
ENSG00000127507 ENSG00000127603 ENSG00000127947 ENSG00000128159 ENSG00000128309
ENSG00000128654 ENSG00000128849 ENSG00000128881 ENSG00000128908 ENSG00000128965
ENSG00000129003 ENSG00000129084 ENSG00000129116 ENSG00000129204 ENSG00000129351
ENSG00000129474 ENSG00000129484 ENSG00000129518 ENSG00000129696 ENSG00000130005
ENSG00000130227 ENSG00000130313 ENSG00000130340 ENSG00000130653 ENSG00000130720

ENSG00000130803	ENSG00000130813	ENSG00000130856	ENSG00000131016	ENSG00000131203
ENSG00000131238	ENSG00000131373	ENSG00000131467	ENSG00000131507	ENSG00000131626
ENSG00000131773	ENSG00000131981	ENSG00000132274	ENSG00000132424	ENSG00000132485
ENSG00000132549	ENSG00000132823	ENSG00000132963	ENSG00000133105	ENSG00000133112
ENSG00000133114	ENSG00000133317	ENSG00000133422	ENSG00000133640	ENSG00000133678
ENSG00000133687	ENSG00000133740	ENSG00000133805	ENSG00000133858	ENSG00000134056
ENSG00000134077	ENSG00000134109	ENSG00000134160	ENSG00000134201	ENSG00000134222
ENSG00000134242	ENSG00000134265	ENSG00000134285	ENSG00000134291	ENSG00000134371
ENSG00000134452	ENSG00000134531	ENSG00000134574	ENSG00000134759	ENSG00000134815
ENSG00000134899	ENSG00000134910	ENSG00000134982	ENSG00000135046	ENSG00000135049
ENSG00000135205	ENSG00000135249	ENSG00000135365	ENSG00000135407	ENSG00000135473
ENSG00000135624	ENSG00000135720	ENSG00000135723	ENSG00000135749	ENSG00000135763
ENSG00000135913	ENSG00000135932	ENSG00000135968	ENSG00000136040	ENSG00000136052
ENSG00000136141	ENSG00000136152	ENSG00000136153	ENSG00000136205	ENSG00000136240
ENSG00000136247	ENSG00000136436	ENSG00000136631	ENSG00000136689	ENSG00000136819
ENSG00000136842	ENSG00000136861	ENSG00000136895	ENSG00000136925	ENSG00000136935
ENSG00000137055	ENSG00000137074	ENSG00000137076	ENSG00000137259	ENSG00000137343
ENSG00000137497	ENSG00000137601	ENSG00000137726	ENSG00000137804	ENSG00000137809
ENSG00000137845	ENSG00000137872	ENSG00000137944	ENSG00000138002	ENSG00000138041
ENSG00000138050	ENSG00000138061	ENSG00000138074	ENSG00000138246	ENSG00000138363
ENSG00000138378	ENSG00000138439	ENSG00000138449	ENSG00000138640	ENSG00000138642
ENSG00000138658	ENSG00000138685	ENSG00000138735	ENSG00000138750	ENSG00000138758
ENSG00000138802	ENSG00000138829	ENSG00000139131	ENSG00000139178	ENSG00000139197
ENSG00000139211	ENSG00000139370	ENSG00000139684	ENSG00000139687	ENSG00000139697
ENSG00000139974	ENSG00000140199	ENSG00000140403	ENSG00000140455	ENSG00000140525
ENSG00000140527	ENSG00000140961	ENSG00000141258	ENSG00000141337	ENSG00000141385
ENSG00000141447	ENSG00000141448	ENSG00000141540	ENSG00000141627	ENSG00000141720
ENSG00000141956	ENSG00000141968	ENSG00000142188	ENSG00000142687	ENSG00000142733
ENSG00000142798	ENSG00000142945	ENSG00000143033	ENSG00000143297	ENSG00000143382
ENSG00000143469	ENSG00000143514	ENSG00000143643	ENSG00000143748	ENSG00000143756
ENSG00000143776	ENSG00000143819	ENSG00000143924	ENSG00000143970	ENSG00000144283
ENSG00000144320	ENSG00000144426	ENSG00000144445	ENSG00000144644	ENSG00000144711
ENSG00000144747	ENSG00000144824	ENSG00000144852	ENSG00000145194	ENSG00000145244
ENSG00000145335	ENSG00000145348	ENSG00000145703	ENSG00000145975	ENSG00000146197
ENSG00000146263	ENSG00000146360	ENSG00000146416	ENSG00000146833	ENSG00000147168
ENSG00000147459	ENSG00000147548	ENSG00000148143	ENSG00000148180	ENSG00000148200
ENSG00000148204	ENSG00000148219	ENSG00000148346	ENSG00000148356	ENSG00000148358
ENSG00000148498	ENSG00000149269	ENSG00000149308	ENSG00000149311	ENSG00000149636
ENSG00000149654	ENSG00000150401	ENSG00000150540	ENSG00000150760	ENSG00000150990
ENSG00000151012	ENSG00000151240	ENSG00000151292	ENSG00000151461	ENSG00000151491
ENSG00000151503	ENSG00000151650	ENSG00000151655	ENSG00000151690	ENSG00000151718
ENSG00000151726	ENSG00000151779	ENSG00000151789	ENSG00000151882	ENSG00000151917
ENSG00000151923	ENSG00000152061	ENSG00000152242	ENSG00000152270	ENSG00000152503
ENSG00000152953	ENSG00000153113	ENSG00000153140	ENSG00000153208	ENSG00000153310
ENSG00000153339	ENSG00000153827	ENSG00000153904	ENSG00000153922	ENSG00000154027
ENSG00000154265	ENSG00000154380	ENSG00000154655	ENSG00000154721	ENSG00000155252
ENSG00000155508	ENSG00000155749	ENSG00000155893	ENSG00000156052	ENSG00000156414
ENSG00000156471	ENSG00000156502	ENSG00000156504	ENSG00000156787	ENSG00000156795
ENSG00000157578	ENSG00000157827	ENSG00000158486	ENSG00000158859	ENSG00000159217
ENSG00000159314	ENSG00000159322	ENSG00000159335	ENSG00000159459	ENSG00000159496
ENSG00000159579	ENSG00000159640	ENSG00000160014	ENSG00000160111	ENSG00000160185

ENSG00000160190 ENSG00000160193 ENSG00000160218 ENSG00000160305 ENSG00000161249
ENSG00000161547 ENSG00000162148 ENSG00000162341 ENSG00000162378 ENSG00000162434
ENSG00000162441 ENSG00000162592 ENSG00000162599 ENSG00000162631 ENSG00000162641
ENSG00000162869 ENSG00000163001 ENSG00000163050 ENSG00000163161 ENSG00000163207
ENSG00000163312 ENSG00000163354 ENSG00000163359 ENSG00000163406 ENSG00000163491
ENSG00000163516 ENSG00000163539 ENSG00000163565 ENSG00000163606 ENSG00000163629
ENSG00000163702 ENSG00000163736 ENSG00000163755 ENSG00000163781 ENSG00000163808
ENSG00000163814 ENSG00000163820 ENSG00000163872 ENSG00000163902 ENSG00000163938
ENSG00000163958 ENSG00000164002 ENSG00000164010 ENSG00000164038 ENSG00000164040
ENSG00000164068 ENSG00000164073 ENSG00000164116 ENSG00000164172 ENSG00000164188
ENSG00000164237 ENSG00000164327 ENSG00000164414 ENSG00000164576 ENSG00000164603
ENSG00000164733 ENSG00000165288 ENSG00000165338 ENSG00000165355 ENSG00000165434
ENSG00000165487 ENSG00000165659 ENSG00000165724 ENSG00000165732 ENSG00000166002
ENSG00000166046 ENSG00000166091 ENSG00000166105 ENSG00000166128 ENSG00000166140
ENSG00000166171 ENSG00000166224 ENSG00000166233 ENSG00000166321 ENSG00000166377
ENSG00000166401 ENSG00000166415 ENSG00000166510 ENSG00000166548 ENSG00000166575
ENSG00000166669 ENSG00000166689 ENSG00000166783 ENSG00000166823 ENSG00000166839
ENSG00000166889 ENSG00000166912 ENSG00000167110 ENSG00000167257 ENSG00000167325
ENSG00000167645 ENSG00000167670 ENSG00000167705 ENSG00000167711 ENSG00000167740
ENSG00000167768 ENSG00000167842 ENSG00000167978 ENSG00000167992 ENSG00000167994
ENSG00000168014 ENSG00000168036 ENSG00000168172 ENSG00000168214 ENSG00000168259
ENSG00000168280 ENSG00000168297 ENSG00000168389 ENSG00000168461 ENSG00000168477
ENSG00000168778 ENSG00000168785 ENSG00000169379 ENSG00000169567 ENSG00000169592
ENSG00000169679 ENSG00000169696 ENSG00000169826 ENSG00000169840 ENSG00000169902
ENSG00000169967 ENSG00000170004 ENSG00000170017 ENSG00000170037 ENSG00000170085
ENSG00000170185 ENSG00000170271 ENSG00000170445 ENSG00000170502 ENSG00000170525
ENSG00000170584 ENSG00000170615 ENSG00000170633 ENSG00000171067 ENSG00000171148
ENSG00000171435 ENSG00000171490 ENSG00000171502 ENSG00000171608 ENSG00000171634
ENSG00000171747 ENSG00000171811 ENSG00000171824 ENSG00000171840 ENSG00000171954
ENSG00000172006 ENSG00000172071 ENSG00000172159 ENSG00000172260 ENSG00000172780
ENSG00000172794 ENSG00000172845 ENSG00000172878 ENSG00000172888 ENSG00000172915
ENSG00000173258 ENSG00000173473 ENSG00000173575 ENSG00000173812 ENSG00000173826
ENSG00000174080 ENSG00000174132 ENSG00000174227 ENSG00000174238 ENSG00000174485
ENSG00000174738 ENSG00000174799 ENSG00000174837 ENSG00000174842 ENSG00000174943
ENSG00000175104 ENSG00000175155 ENSG00000175198 ENSG00000175216 ENSG00000175224
ENSG00000175294 ENSG00000175471 ENSG00000175718 ENSG00000175931 ENSG00000176208
ENSG00000176531 ENSG00000176623 ENSG00000177058 ENSG00000177103 ENSG00000177200
ENSG00000177324 ENSG00000177380 ENSG00000177565 ENSG00000177595 ENSG00000177640
ENSG00000177646 ENSG00000177932 ENSG00000178035 ENSG00000178202 ENSG00000178397
ENSG00000179152 ENSG00000179213 ENSG00000179262 ENSG00000179348 ENSG00000180264
ENSG00000180488 ENSG00000180532 ENSG00000180628 ENSG00000180871 ENSG00000181652
ENSG00000181786 ENSG00000181826 ENSG00000181827 ENSG00000182208 ENSG00000182512
ENSG00000182871 ENSG00000182934 ENSG00000183023 ENSG00000183091 ENSG00000183291
ENSG00000183486 ENSG00000183495 ENSG00000183576 ENSG00000183605 ENSG00000183690
ENSG00000183914 ENSG00000183963 ENSG00000184014 ENSG00000184156 ENSG00000184221
ENSG00000184634 ENSG00000184752 ENSG00000184792 ENSG00000184922 ENSG00000185046
ENSG00000185246 ENSG00000185418 ENSG00000185480 ENSG00000185630 ENSG00000186001
ENSG00000186073 ENSG00000186104 ENSG00000186141 ENSG00000186166 ENSG00000186409
ENSG00000186448 ENSG00000186529 ENSG00000186648 ENSG00000186815 ENSG00000186908
ENSG00000186919 ENSG00000187008 ENSG00000187051 ENSG00000187164 ENSG00000187244
ENSG00000187266 ENSG00000187796 ENSG00000187823 ENSG00000187848 ENSG00000188352

ENSG00000188393 ENSG00000189060 ENSG00000189077 ENSG00000189319 ENSG00000196150
ENSG00000196152 ENSG00000196220 ENSG00000196381 ENSG00000196569 ENSG00000196642
ENSG00000196652 ENSG00000196712 ENSG00000196739 ENSG00000196792 ENSG00000196914
ENSG00000196987 ENSG00000197045 ENSG00000197122 ENSG00000197142 ENSG00000197181
ENSG00000197321 ENSG00000197363 ENSG00000197375 ENSG00000197430 ENSG00000197563
ENSG00000197601 ENSG00000197616 ENSG00000197635 ENSG00000197701 ENSG00000197779
ENSG00000197860 ENSG00000197863 ENSG00000197930 ENSG00000197965 ENSG00000197971
ENSG00000198001 ENSG00000198130 ENSG00000198162 ENSG00000198231 ENSG00000198315
ENSG00000198326 ENSG00000198431 ENSG00000198466 ENSG00000198556 ENSG00000198561
ENSG00000198673 ENSG00000198690 ENSG00000198719 ENSG00000198740 ENSG00000198752
ENSG00000198818 ENSG00000198835 ENSG00000198959 ENSG00000203965 ENSG00000204130
ENSG00000204219 ENSG00000204385 ENSG00000204410 ENSG00000204472 ENSG00000204613
ENSG00000204681 ENSG00000204815 ENSG00000204843 ENSG00000205683 ENSG00000205981
ENSG00000206560 ENSG00000211456 ENSG00000211890 ENSG00000213022 ENSG00000213185
ENSG00000213920 ENSG00000213983 ENSG00000214078 ENSG00000215193 ENSG00000215301
ENSG00000215440 ENSG00000221823 ENSG00000221826 ENSG00000221829 ENSG00000221914
ENSG00000228175 ENSG00000228340 ENSG00000230626 ENSG00000231925 ENSG00000236699
ENSG00000239521 ENSG00000239900 ENSG00000241697 ENSG00000241852 ENSG00000241878
ENSG00000241973 ENSG00000242950 ENSG00000243811 ENSG00000244687 ENSG00000248483
ENSG00000249087 ENSG00000249978 ENSG00000254093 ENSG00000255883 ENSG00000256043
ENSG00000256660 ENSG00000256771 ENSG00000257335 ENSG00000258128 ENSG00000258890

6.2 Lower Inclusion in Cases

ENSG00000000971 ENSG00000001036 ENSG00000003400 ENSG00000003989 ENSG00000004660
ENSG00000005381 ENSG00000005436 ENSG00000008441 ENSG00000011600 ENSG00000013306
ENSG00000015475 ENSG00000021762 ENSG00000023839 ENSG00000025796 ENSG00000028116
ENSG00000028203 ENSG00000032742 ENSG00000037757 ENSG00000042832 ENSG00000046651
ENSG00000047597 ENSG00000049192 ENSG00000049759 ENSG00000050393 ENSG00000055208
ENSG00000056972 ENSG00000057252 ENSG00000058272 ENSG00000059769 ENSG00000059915
ENSG00000064012 ENSG00000064102 ENSG00000064270 ENSG00000064961 ENSG00000065328
ENSG00000065534 ENSG00000065809 ENSG00000066294 ENSG00000068784 ENSG00000068885
ENSG00000069535 ENSG00000070182 ENSG00000070444 ENSG00000070610 ENSG00000074803
ENSG00000075891 ENSG00000075945 ENSG00000078618 ENSG00000079308 ENSG00000080007
ENSG00000080561 ENSG00000082458 ENSG00000083097 ENSG00000083520 ENSG00000083544
ENSG00000084234 ENSG00000084774 ENSG00000086232 ENSG00000086506 ENSG00000086717
ENSG00000087586 ENSG00000088726 ENSG00000089091 ENSG00000090238 ENSG00000091039
ENSG00000091128 ENSG00000091181 ENSG00000092421 ENSG00000092758 ENSG00000093167
ENSG00000093217 ENSG00000094914 ENSG00000095321 ENSG00000096696 ENSG00000097046
ENSG00000100023 ENSG00000100225 ENSG00000100325 ENSG00000100441 ENSG00000100490
ENSG00000101004 ENSG00000101134 ENSG00000101670 ENSG00000101773 ENSG00000101782
ENSG00000102189 ENSG00000102313 ENSG00000102471 ENSG00000102755 ENSG00000103148
ENSG00000103540 ENSG00000103723 ENSG00000104903 ENSG00000105173 ENSG00000105255
ENSG00000105270 ENSG00000105613 ENSG00000105929 ENSG00000106771 ENSG00000106804
ENSG00000106853 ENSG00000107338 ENSG00000107951 ENSG00000108309 ENSG00000108344
ENSG00000108576 ENSG00000108666 ENSG00000108852 ENSG00000108861 ENSG00000109182
ENSG00000109458 ENSG00000109625 ENSG00000109680 ENSG00000110063 ENSG00000110274
ENSG00000110811 ENSG00000111319 ENSG00000111530 ENSG00000111581 ENSG00000111602
ENSG00000111700 ENSG00000111846 ENSG00000111879 ENSG00000112210 ENSG00000112293
ENSG00000112305 ENSG00000112335 ENSG00000112414 ENSG00000112494 ENSG00000112761
ENSG00000112769 ENSG00000112983 ENSG00000113140 ENSG00000113456 ENSG00000114656

ENSG00000114956 ENSG00000115020 ENSG00000115145 ENSG00000115419 ENSG00000115425
ENSG00000115841 ENSG00000116254 ENSG00000116285 ENSG00000116711 ENSG00000116748
ENSG00000117115 ENSG00000117592 ENSG00000118434 ENSG00000119041 ENSG00000119283
ENSG00000119684 ENSG00000119782 ENSG00000119919 ENSG00000119927 ENSG00000120725
ENSG00000120727 ENSG00000120899 ENSG00000121039 ENSG00000121075 ENSG00000121101
ENSG00000122025 ENSG00000122299 ENSG00000122591 ENSG00000122783 ENSG00000123500
ENSG00000123572 ENSG00000124092 ENSG00000124571 ENSG00000125037 ENSG00000125207
ENSG00000125388 ENSG00000125975 ENSG00000126777 ENSG00000127955 ENSG00000128918
ENSG00000129003 ENSG00000129295 ENSG00000129472 ENSG00000129646 ENSG00000130158
ENSG00000130433 ENSG00000130813 ENSG00000131023 ENSG00000131203 ENSG00000131437
ENSG00000131626 ENSG00000132330 ENSG00000132535 ENSG00000132623 ENSG00000132780
ENSG00000132819 ENSG00000132823 ENSG00000132854 ENSG00000133107 ENSG00000133134
ENSG00000133678 ENSG00000133858 ENSG00000133997 ENSG00000134265 ENSG00000134321
ENSG00000134668 ENSG00000134765 ENSG00000134853 ENSG00000135049 ENSG00000135632
ENSG00000135862 ENSG00000135945 ENSG00000135951 ENSG00000136051 ENSG00000136819
ENSG00000136828 ENSG00000136842 ENSG00000136929 ENSG00000137210 ENSG00000137502
ENSG00000137804 ENSG00000137809 ENSG00000137815 ENSG00000138115 ENSG00000138119
ENSG00000138246 ENSG00000138448 ENSG00000138641 ENSG00000138722 ENSG00000139219
ENSG00000139220 ENSG00000139287 ENSG00000139915 ENSG00000140015 ENSG00000140403
ENSG00000140416 ENSG00000140525 ENSG00000140795 ENSG00000140798 ENSG00000141012
ENSG00000141447 ENSG00000141503 ENSG00000141720 ENSG00000142230 ENSG00000142973
ENSG00000143341 ENSG00000143416 ENSG00000143420 ENSG00000143552 ENSG00000143740
ENSG00000144063 ENSG00000144554 ENSG00000144668 ENSG00000144674 ENSG00000144749
ENSG00000144935 ENSG00000145335 ENSG00000145703 ENSG00000145730 ENSG00000145850
ENSG00000146463 ENSG00000146555 ENSG00000146700 ENSG00000147459 ENSG00000149091
ENSG00000149452 ENSG00000149485 ENSG00000149577 ENSG00000150048 ENSG00000150471
ENSG00000150667 ENSG00000151353 ENSG00000151461 ENSG00000151490 ENSG00000151491
ENSG00000151655 ENSG00000151929 ENSG00000152061 ENSG00000152782 ENSG00000153208
ENSG00000154114 ENSG00000154330 ENSG00000154832 ENSG00000157330 ENSG00000157827
ENSG00000158106 ENSG00000158301 ENSG00000158481 ENSG00000158856 ENSG00000158865
ENSG00000159023 ENSG00000159314 ENSG00000159348 ENSG00000159588 ENSG00000160199
ENSG00000160345 ENSG00000160746 ENSG00000160808 ENSG00000161791 ENSG00000162383
ENSG00000162706 ENSG00000162753 ENSG00000162882 ENSG00000162971 ENSG00000162999
ENSG00000163006 ENSG00000163093 ENSG00000163141 ENSG00000163467 ENSG00000163491
ENSG00000163751 ENSG00000163958 ENSG00000164010 ENSG00000164116 ENSG00000164168
ENSG00000164237 ENSG00000164733 ENSG00000164794 ENSG00000165521 ENSG00000165525
ENSG00000165682 ENSG00000165684 ENSG00000165995 ENSG00000166147 ENSG00000166200
ENSG00000166704 ENSG00000166783 ENSG00000167671 ENSG00000167705 ENSG00000167723
ENSG00000167741 ENSG00000167800 ENSG00000167971 ENSG00000168268 ENSG00000168439
ENSG00000168481 ENSG00000168785 ENSG00000168904 ENSG00000169118 ENSG00000169251
ENSG00000169418 ENSG00000170271 ENSG00000170417 ENSG00000170502 ENSG00000170899
ENSG00000171033 ENSG00000171219 ENSG00000171243 ENSG00000171298 ENSG00000171503
ENSG00000171723 ENSG00000172260 ENSG00000172915 ENSG00000173276 ENSG00000173530
ENSG00000174080 ENSG00000174125 ENSG00000174672 ENSG00000175155 ENSG00000175216
ENSG00000175600 ENSG00000175832 ENSG00000176153 ENSG00000176236 ENSG00000176406
ENSG00000176998 ENSG00000177108 ENSG00000178026 ENSG00000178075 ENSG00000178096
ENSG00000179262 ENSG00000179361 ENSG00000179914 ENSG00000183091 ENSG00000183160
ENSG00000183378 ENSG00000183508 ENSG00000183605 ENSG00000183625 ENSG00000183844
ENSG00000184083 ENSG00000184258 ENSG00000184408 ENSG00000184445 ENSG00000184752
ENSG00000184792 ENSG00000185418 ENSG00000185532 ENSG00000185909 ENSG00000186074
ENSG00000186417 ENSG00000186431 ENSG00000186918 ENSG00000186976 ENSG00000187391

ENSG00000187475 ENSG00000187792 ENSG00000187889 ENSG00000188419 ENSG00000188580
ENSG00000188869 ENSG00000189060 ENSG00000189108 ENSG00000196914 ENSG00000196975
ENSG00000197241 ENSG00000197275 ENSG00000197343 ENSG00000197635 ENSG00000197701
ENSG00000197771 ENSG00000198369 ENSG00000198561 ENSG00000198626 ENSG00000198718
ENSG00000198795 ENSG00000198818 ENSG00000198846 ENSG00000198873 ENSG00000203965
ENSG00000203995 ENSG00000204410 ENSG00000204420 ENSG00000204611 ENSG00000204655
ENSG00000204852 ENSG00000205744 ENSG00000206190 ENSG00000211593 ENSG00000213339
ENSG00000214174 ENSG00000214397 ENSG00000214655 ENSG00000214944 ENSG00000215483
ENSG00000221968 ENSG00000230701 ENSG00000237506 ENSG00000239282 ENSG00000243667
ENSG00000247626 ENSG00000250722 ENSG00000256269 ENSG00000257151 ENSG00000259753

7 Associated Exons

7.1 Higher Inclusion in Cases

The list of exons analysed represent a one-to-one mapping of probesets to exons (see *Methods*). The set of exons shown here represent a random choice of all possible exons that overlap with the exon array probeset. The number after the colon (':') represents the exon number with the transcript isoform.

ENST00000005340:4 ENST00000011700:2
ENST00000013125:29 ENST00000025301:2
ENST00000040877:23 ENST00000075503:8
ENST00000082259:9 ENST00000083182:7
ENST00000158149:10 ENST00000158771:2
ENST00000164133:13 ENST00000164133:5
ENST00000198765:9 ENST00000200135:14
ENST00000200135:5 ENST00000202017:4
ENST00000202816:5 ENST00000207549:16
ENST00000210060:8 ENST00000215862:19
ENST00000216115:4 ENST00000216492:6
ENST00000217800:7 ENST00000217909:4
ENST00000219070:4 ENST00000221973:2
ENST00000223642:40 ENST00000225308:3
ENST00000229214:5 ENST00000229729:17
ENST00000230568:2 ENST00000230901:15
ENST00000231368:11 ENST00000234420:4
ENST00000236959:12 ENST00000237186:4
ENST00000237612:5 ENST00000237612:8
ENST00000238831:3 ENST00000242057:7
ENST00000242210:4 ENST00000242351:9
ENST00000243346:3 ENST00000243346:7
ENST00000243583:13 ENST00000243662:2
ENST00000243903:7 ENST00000244426:9
ENST00000244769:6 ENST00000246314:2
ENST00000247400:4 ENST00000250572:6
ENST00000251108:3 ENST00000251900:7
ENST00000252603:4 ENST00000253159:23
ENST00000253814:5 ENST00000254442:14
ENST00000254821:3 ENST00000256117:6
ENST00000257931:20 ENST00000258243:8
ENST00000258526:12 ENST00000258538:9
ENST00000260408:5 ENST00000260508:12
ENST00000260818:42 ENST00000261207:17
ENST00000261405:39 ENST00000261483:15
ENST00000261483:17 ENST00000261483:19
ENST00000261483:4 ENST00000261517:56
ENST00000261530:7 ENST00000261584:10
ENST00000261667:10 ENST00000261681:5
ENST00000261755:9 ENST00000261867:6
ENST00000262134:5 ENST00000262262:2
ENST00000262352:8 ENST00000262455:2
ENST00000262464:61 ENST00000262539:17
ENST00000262738:27 ENST00000263119:31
ENST00000263253:20 ENST00000263354:6
ENST00000263441:11 ENST00000263574:5
ENST00000263636:10 ENST00000263754:3
ENST00000263802:13 ENST00000263956:16
ENST00000263962:3 ENST00000264167:17
ENST00000264331:9 ENST00000264436:15
ENST00000264498:2 ENST00000264646:10
ENST00000264705:10 ENST00000264705:24
ENST00000264805:19 ENST00000264825:9
ENST00000264827:16 ENST00000264951:4
ENST00000265062:5 ENST00000265081:11
ENST00000265382:11 ENST00000265872:16
ENST00000266041:8 ENST00000267163:15
ENST00000268130:5 ENST00000269216:5
ENST00000269445:4 ENST00000269703:4
ENST00000274327:9 ENST00000274364:12

ENST00000274938:15 ENST00000275053:19
ENST00000276440:46 ENST00000277531:13
ENST00000278568:10 ENST00000278612:15
ENST00000278616:31 ENST00000280333:45
ENST00000280612:3 ENST00000281513:18
ENST00000281772:9 ENST00000282007:8
ENST00000282059:3 ENST00000282096:2
ENST00000282538:8 ENST00000283351:17
ENST00000283351:6 ENST00000283684:15
ENST00000284031:2 ENST00000284049:24
ENST00000286653:25 ENST00000290341:8
ENST00000290650:43 ENST00000291574:5
ENST00000294309:9 ENST00000294600:5
ENST00000296129:2 ENST00000296137:5
ENST00000296446:4 ENST00000296658:5
ENST00000298097:2 ENST00000298198:10
ENST00000298585:5 ENST00000299045:9
ENST00000299167:30 ENST00000299297:3
ENST00000300417:15 ENST00000300650:2
ENST00000301336:5 ENST00000301843:9
ENST00000303372:8 ENST00000303375:20
ENST00000303375:27 ENST00000303965:25
ENST00000304076:8 ENST00000304457:7
ENST00000305494:14 ENST00000306729:7
ENST00000307602:19 ENST00000307751:4
ENST00000308736:2 ENST00000309336:9
ENST00000310775:15 ENST00000310775:35
ENST00000311500:3 ENST00000312637:2
ENST00000312655:3 ENST00000312858:6
ENST00000313663:26 ENST00000314823:2
ENST00000315475:4 ENST00000315596:26
ENST00000315796:7 ENST00000316219:6
ENST00000316856:5 ENST00000317147:7
ENST00000317243:4 ENST00000317296:2
ENST00000317677:5 ENST00000318522:12
ENST00000319380:7 ENST00000319481:22
ENST00000321169:12 ENST00000321322:4
ENST00000321370:23 ENST00000321990:18
ENST00000322349:19 ENST00000323441:11
ENST00000323456:17 ENST00000324219:10
ENST00000324696:19 ENST00000325674:20
ENST00000325722:19 ENST00000325980:2
ENST00000326080:6 ENST00000326080:8
ENST00000326682:2 ENST00000327087:3
ENST00000328118:11 ENST00000329466:13
ENST00000330236:2 ENST00000331768:4
ENST00000333577:2 ENST00000333807:3
ENST00000334186:8 ENST00000334318:2
ENST00000334420:35 ENST00000334810:19
ENST00000334859:3 ENST00000335387:3
ENST00000335742:17 ENST00000336332:3
ENST00000336735:3 ENST00000337006:8
ENST00000337318:3 ENST00000337679:3
ENST00000338608:6 ENST00000339497:5
ENST00000339766:3 ENST00000340022:18
ENST00000340989:17 ENST00000341950:2
ENST00000342160:80 ENST00000342505:13
ENST00000342905:6 ENST00000342981:7
ENST00000343439:8 ENST00000344492:4
ENST00000344754:12 ENST00000344908:3
ENST00000345102:2 ENST00000345990:10
ENST00000346432:5 ENST00000348141:3
ENST00000348335:4 ENST00000348616:5
ENST00000348925:2 ENST00000348959:9
ENST00000349456:2 ENST00000349995:7
ENST00000352131:9 ENST00000352171:17
ENST00000352171:25 ENST00000353578:10
ENST00000354185:3 ENST00000354241:3
ENST00000354273:10 ENST00000354379:3
ENST00000354417:3 ENST00000354558:5
ENST00000354747:7 ENST00000354831:21
ENST00000355221:2 ENST00000355674:5
ENST00000355725:17 ENST00000355758:6
ENST00000355824:4 ENST00000355867:7
ENST00000356081:2 ENST00000356083:43
ENST00000356248:7 ENST00000356287:36
ENST00000356462:15 ENST00000356592:4
ENST00000356641:26 ENST00000356644:7
ENST00000357103:2 ENST00000357321:2
ENST00000357349:12 ENST00000357387:19
ENST00000357395:27 ENST00000357474:6
ENST00000357482:6 ENST00000357563:6
ENST00000357602:10 ENST00000357604:10
ENST00000357637:10 ENST00000357895:20
ENST00000358025:94 ENST00000358032:5
ENST00000358136:3 ENST00000358499:6
ENST00000358544:23 ENST00000358552:8
ENST00000358582:2 ENST00000358675:2
ENST00000358951:20 ENST00000359142:18
ENST00000359652:14 ENST00000359684:7
ENST00000359785:7 ENST00000360222:15
ENST00000360385:2 ENST00000360822:17
ENST00000361078:14 ENST00000361246:8
ENST00000361354:18 ENST00000361477:21
ENST00000361689:10 ENST00000361731:2
ENST00000361937:4 ENST00000366756:3
ENST00000366920:22 ENST00000366922:15
ENST00000366976:4 ENST00000367079:8
ENST00000367350:19 ENST00000367429:13
ENST00000367435:10 ENST00000367472:10
ENST00000367506:13 ENST00000367585:2
ENST00000367591:9 ENST00000367996:4

ENST00000368033:5 ENST00000368132:6
ENST00000368305:2 ENST00000368331:7
ENST00000368346:8 ENST00000368876:8
ENST00000368898:3 ENST00000369039:17
ENST00000369294:3 ENST00000369556:2
ENST00000369903:3 ENST00000369936:7
ENST00000369983:8 ENST00000370343:12
ENST00000370420:11 ENST00000370513:19
ENST00000370513:3 ENST00000370631:7
ENST00000370787:2 ENST00000370958:4
ENST00000371184:4 ENST00000371189:5
ENST00000371477:7 ENST00000371805:10
ENST00000371936:3 ENST00000372258:8
ENST00000372406:17 ENST00000372410:4
ENST00000372447:3 ENST00000372883:33
ENST00000372899:36 ENST00000372901:44
ENST00000372970:4 ENST00000373017:6
ENST00000373365:2 ENST00000373555:15
ENST00000373558:2 ENST00000373584:3
ENST00000373620:4 ENST00000373750:5
ENST00000373823:21 ENST00000374152:8
ENST00000374464:4 ENST00000374512:10
ENST00000374695:46 ENST00000374776:8
ENST00000374864:13 ENST00000374879:6
ENST00000374879:9 ENST00000375165:4
ENST00000375221:15 ENST00000375226:101
ENST00000375247:40 ENST00000375750:4
ENST00000375955:13 ENST00000376277:17
ENST00000376485:2 ENST00000376704:3
ENST00000376740:4 ENST00000376872:20
ENST00000376887:19 ENST00000376887:20
ENST00000377223:4 ENST00000377540:6
ENST00000377826:9 ENST00000377974:11
ENST00000378128:6 ENST00000378164:6
ENST00000378601:2 ENST00000378616:2
ENST00000378643:5 ENST00000378697:6
ENST00000378715:7 ENST00000378722:4
ENST00000378748:11 ENST00000379028:11
ENST00000379120:9 ENST00000379374:8
ENST00000379568:5 ENST00000379587:15
ENST00000379868:5 ENST00000379939:34
ENST00000379965:6 ENST00000380030:7
ENST00000380033:13 ENST00000380249:14
ENST00000380314:14 ENST00000380358:31
ENST00000380649:4 ENST00000380671:7
ENST00000380737:9 ENST00000380746:18
ENST00000381007:7 ENST00000381340:21
ENST00000381340:6 ENST00000381938:5
ENST00000381989:17 ENST00000381992:12
ENST00000382058:3 ENST00000382850:8
ENST00000382891:3 ENST00000383744:4
ENST00000383771:11 ENST00000388768:9
ENST00000389044:32 ENST00000389720:5
ENST00000389721:9 ENST00000389722:16
ENST00000391955:10 ENST00000392045:4
ENST00000392257:4 ENST00000392257:6
ENST00000392320:9 ENST00000392465:2
ENST00000392529:7 ENST00000392614:5
ENST00000392677:3 ENST00000392717:8
ENST00000392901:10 ENST00000393308:2
ENST00000393729:15 ENST00000394060:3
ENST00000394063:5 ENST00000394606:2
ENST00000394821:14 ENST00000394822:6
ENST00000394822:9 ENST00000394886:8
ENST00000395125:20 ENST00000395211:6
ENST00000395308:5 ENST00000395841:2
ENST00000395886:6 ENST00000395896:49
ENST00000395898:10 ENST00000395911:7
ENST00000395996:31 ENST00000396031:10
ENST00000396234:16 ENST00000396281:19
ENST00000396328:15 ENST00000396658:2
ENST00000396676:7 ENST00000396706:4
ENST00000396865:11 ENST00000397227:3
ENST00000397311:9 ENST00000397736:6
ENST00000397881:2 ENST00000398129:10
ENST00000398208:3 ENST00000398341:11
ENST00000398367:11 ENST00000398569:38
ENST00000398569:44 ENST00000398835:5
ENST00000399100:7 ENST00000399100:8
ENST00000399166:8 ENST00000399799:26
ENST00000399918:3 ENST00000400105:15
ENST00000400334:25 ENST00000400345:18
ENST00000400537:6 ENST00000401461:11
ENST00000401475:11 ENST00000401979:14
ENST00000402569:3 ENST00000402938:7
ENST00000403733:12 ENST00000404125:23
ENST00000404251:10 ENST00000404251:7
ENST00000404424:2 ENST00000404816:27
ENST00000405383:4 ENST00000405570:12
ENST00000406875:3 ENST00000409311:6
ENST00000409788:5 ENST00000411829:12
ENST00000412096:5 ENST00000414226:9
ENST00000415096:14 ENST00000415178:51
ENST00000415402:8 ENST00000415482:3
ENST00000415485:19 ENST00000415511:2
ENST00000415570:7 ENST00000415740:4
ENST00000415849:3 ENST00000415948:5
ENST00000416445:9 ENST00000417408:8
ENST00000417413:2 ENST00000417641:13
ENST00000417768:6 ENST00000418019:11
ENST00000418019:22 ENST00000418390:2
ENST00000418472:11 ENST00000418984:15

ENST00000419091:6 ENST00000419286:2
ENST00000419479:5 ENST00000419504:22
ENST00000419533:13 ENST00000420083:2
ENST00000420166:3 ENST00000420632:9
ENST00000420886:29 ENST00000420906:9
ENST00000421084:27 ENST00000421317:7
ENST00000421344:8 ENST00000421660:28
ENST00000421748:6 ENST00000421794:4
ENST00000421865:23 ENST00000422063:6
ENST00000422431:7 ENST00000423109:2
ENST00000423760:10 ENST00000424123:4
ENST00000424254:2 ENST00000424848:6
ENST00000425217:19 ENST00000425518:16
ENST00000426203:2 ENST00000426496:9
ENST00000426588:10 ENST00000426639:46
ENST00000426726:2 ENST00000426778:10
ENST00000426855:21 ENST00000427159:8
ENST00000427231:78 ENST00000428210:9
ENST00000428532:12 ENST00000429687:13
ENST00000430069:13 ENST00000430153:7
ENST00000430906:8 ENST00000430930:25
ENST00000430930:28 ENST00000431156:3
ENST00000431614:11 ENST00000431932:22
ENST00000432185:5 ENST00000432910:4
ENST00000433067:5 ENST00000433336:7
ENST00000433384:4 ENST00000433477:5
ENST00000433566:10 ENST00000434243:6
ENST00000434583:9 ENST00000434918:25
ENST00000434949:2 ENST00000435252:2
ENST00000435944:2 ENST00000435957:2
ENST00000437205:2 ENST00000438053:2
ENST00000438563:3 ENST00000439966:13
ENST00000440591:5 ENST00000441077:2
ENST00000441946:7 ENST00000442506:21
ENST00000442913:7 ENST00000443236:19
ENST00000443236:22 ENST00000443351:6
ENST00000443617:22 ENST00000443617:62
ENST00000443617:74 ENST00000443751:3
ENST00000444139:4 ENST00000444181:3
ENST00000444189:2 ENST00000444746:27
ENST00000444746:8 ENST00000444851:3
ENST00000444971:3 ENST00000445230:2
ENST00000445992:3 ENST00000446066:9
ENST00000446119:3 ENST00000446172:3
ENST00000446394:15 ENST00000446688:7
ENST00000446688:8 ENST00000446688:9
ENST00000447540:24 ENST00000447611:13
ENST00000447614:8 ENST00000448202:3
ENST00000448504:3 ENST00000449213:3
ENST00000449802:48 ENST00000450318:9
ENST00000450444:7 ENST00000450465:4
ENST00000450617:11 ENST00000451779:5
ENST00000452508:22 ENST00000452837:7
ENST00000453024:13 ENST00000453621:5
ENST00000453658:13 ENST00000453764:8
ENST00000454158:3 ENST00000455186:6
ENST00000455558:6 ENST00000456735:9
ENST00000456784:3 ENST00000457138:14
ENST00000457188:3 ENST00000457572:4
ENST00000457679:19 ENST00000457800:10
ENST00000457905:14 ENST00000457913:12
ENST00000459737:4 ENST00000460073:2
ENST00000460495:8 ENST00000460658:2
ENST00000461319:2 ENST00000461696:2
ENST00000461774:3 ENST00000463046:9
ENST00000463088:5 ENST00000463496:4
ENST00000463975:3 ENST00000464661:4
ENST00000464941:7 ENST00000464952:2
ENST00000465020:4 ENST00000465445:8
ENST00000465942:5 ENST00000466192:2
ENST00000466205:2 ENST00000466380:4
ENST00000467023:2 ENST00000467183:14
ENST00000467991:3 ENST00000468222:5
ENST00000468286:4 ENST00000469075:15
ENST00000469553:12 ENST00000470408:3
ENST00000470599:2 ENST00000470781:10
ENST00000470890:2 ENST00000470902:4
ENST00000471069:4 ENST00000471302:2
ENST00000471652:7 ENST00000471858:4
ENST00000472232:2 ENST00000472414:5
ENST00000472428:2 ENST00000472433:2
ENST00000473642:2 ENST00000473691:4
ENST00000473795:3 ENST00000474225:3
ENST00000474314:4 ENST00000474606:5
ENST00000474859:4 ENST00000475265:3
ENST00000475443:2 ENST00000475560:6
ENST00000475668:27 ENST00000475675:4
ENST00000476978:2 ENST00000477308:3
ENST00000478407:2 ENST00000478412:3
ENST00000478588:4 ENST00000478777:7
ENST00000479241:13 ENST00000479605:2
ENST00000479877:4 ENST00000480112:24
ENST00000480112:33 ENST00000480281:2
ENST00000480606:2 ENST00000480606:4
ENST00000480623:5 ENST00000480886:3
ENST00000481266:6 ENST00000481518:6
ENST00000481751:2 ENST00000481965:3
ENST00000482064:3 ENST00000482456:22
ENST00000482755:2 ENST00000482953:4
ENST00000483398:4 ENST00000483593:5
ENST00000483871:3 ENST00000483888:33
ENST00000484220:2 ENST00000485566:4

ENST00000485568:6 ENST00000485572:10
ENST00000486040:3 ENST00000486979:12
ENST00000487128:11 ENST00000487200:7
ENST00000487556:5 ENST00000487883:2
ENST00000488010:5 ENST00000488149:25
ENST00000489711:14 ENST00000489740:2
ENST00000490210:6 ENST00000490286:17
ENST00000490566:5 ENST00000490873:6
ENST00000492574:7 ENST00000493041:3
ENST00000494090:26 ENST00000495044:5
ENST00000495360:6 ENST00000495654:3
ENST00000495713:2 ENST00000497002:4
ENST00000497155:6 ENST00000497286:7
ENST00000497558:5 ENST00000497607:3
ENST00000498012:3 ENST00000498077:19
ENST00000498144:6 ENST00000498211:3
ENST00000498611:22 ENST00000498699:10
ENST00000503230:10 ENST00000503868:3
ENST00000504202:4 ENST00000504584:2
ENST00000504706:2 ENST00000504872:4
ENST00000506202:15 ENST00000506733:20
ENST00000506799:5 ENST00000506816:4
ENST00000506828:2 ENST00000507184:28
ENST00000508066:12 ENST00000508465:2
ENST00000508466:10 ENST00000508895:5
ENST00000509667:2 ENST00000509732:2
ENST00000509768:6 ENST00000510377:6
ENST00000510533:11 ENST00000510533:4
ENST00000510573:13 ENST00000510985:12
ENST00000511439:4 ENST00000511939:17
ENST00000512575:2 ENST00000512636:8
ENST00000513031:2 ENST00000513079:4
ENST00000513245:4 ENST00000513695:4
ENST00000513837:8 ENST00000514141:8
ENST00000514154:5 ENST00000515201:6
ENST00000517339:24 ENST00000518211:4
ENST00000519449:4 ENST00000520113:11
ENST00000521134:14 ENST00000521356:4
ENST00000522052:3 ENST00000522072:9
ENST00000522083:3 ENST00000522394:2
ENST00000522420:13 ENST00000522874:2
ENST00000523170:2 ENST00000523212:3
ENST00000523582:17 ENST00000523756:15
ENST00000524114:5 ENST00000525013:3
ENST00000525781:7 ENST00000525964:27
ENST00000525971:11 ENST00000526262:7
ENST00000526995:5 ENST00000526995:6
ENST00000527141:2 ENST00000527587:10
ENST00000527591:9 ENST00000528744:2
ENST00000529244:5 ENST00000529588:4
ENST00000529744:3 ENST00000529970:20
ENST00000530044:2 ENST00000530704:2
ENST00000530879:2 ENST00000531107:19
ENST00000532259:5 ENST00000532502:2
ENST00000532742:2 ENST00000533349:4
ENST00000533801:14 ENST00000534548:12
ENST00000534548:9 ENST00000534738:5
ENST00000534881:11 ENST00000535095:9
ENST00000535106:7 ENST00000535117:4
ENST00000535254:13 ENST00000535346:2
ENST00000535650:11 ENST00000536121:2
ENST00000536256:11 ENST00000536291:4
ENST00000536649:5 ENST00000536681:8
ENST00000536688:10 ENST00000536701:6
ENST00000536756:4 ENST00000536796:17
ENST00000536845:17 ENST00000537036:8
ENST00000537081:8 ENST00000537084:21
ENST00000537174:11 ENST00000537599:11
ENST00000538066:16 ENST00000538611:3
ENST00000539368:8 ENST00000539476:8
ENST00000539513:6 ENST00000539524:29
ENST00000539592:11 ENST00000539625:10
ENST00000539933:3 ENST00000540043:8
ENST00000540216:7 ENST00000540246:3
ENST00000540418:6 ENST00000540444:42
ENST00000540823:7 ENST00000540905:5
ENST00000541133:5 ENST00000541188:8
ENST00000541296:8 ENST00000541377:29
ENST00000541394:10 ENST00000541549:8
ENST00000542094:5 ENST00000542289:2
ENST00000542339:4 ENST00000542445:9
ENST00000542526:15 ENST00000542979:6
ENST00000543127:5 ENST00000543192:11
ENST00000543523:16 ENST00000543645:9
ENST00000543648:2 ENST00000543662:9
ENST00000543949:10 ENST00000544784:7
ENST00000544853:4 ENST00000544933:3
ENST00000545132:6 ENST00000546208:10
ENST00000546893:8 ENST00000547043:7
ENST00000547101:6 ENST00000547372:15
ENST00000547540:2 ENST00000548465:2
ENST00000549049:5 ENST00000549379:5
ENST00000549851:7 ENST00000549944:7
ENST00000550672:5 ENST00000551079:2
ENST00000551673:2 ENST00000552351:7
ENST00000552618:13 ENST00000552641:5
ENST00000552738:23 ENST00000552994:23
ENST00000553217:2 ENST00000553369:5
ENST00000553412:15 ENST00000554164:2
ENST00000554439:2 ENST00000554465:4
ENST00000554715:5 ENST00000554752:9
ENST00000555223:6 ENST00000555358:13

ENST00000556626:19 ENST00000556626:5 ENST00000557018:4 ENST00000557332:9 ENST00000557381:27 ENST00000558157:14 ENST00000558768:20 ENST00000559236:2 ENST00000560792:5 ENST00000561552:4 ENST00000562768:4 ENST00000563197:2 ENST00000563264:3 ENST00000564674:6 ENST00000565267:11 ENST00000565435:3 ENST00000565488:4 ENST00000565502:3 ENST00000565644:3 ENST00000565728:10 ENST00000566389:3 ENST00000566621:3 ENST00000569057:4

7.2 Lower Inclusion in Cases

ENST00000040663:4 ENST00000199708:2 ENST00000218436:8 ENST00000221856:3 ENST00000225275:10 ENST00000225805:8 ENST00000226432:6 ENST00000228916:7 ENST00000231061:9 ENST00000237163:30 ENST00000237201:3 ENST00000243300:26 ENST00000243914:10 ENST00000246199:2 ENST00000251507:10 ENST00000252729:3 ENST00000253110:5 ENST00000255174:3 ENST00000256379:5 ENST00000257100:11 ENST00000257198:4 ENST00000257290:9 ENST00000258341:21 ENST00000259351:2 ENST00000261292:9 ENST00000261707:9 ENST00000261722:18 ENST00000263063:7 ENST00000264345:2 ENST00000264705:2 ENST00000264741:24 ENST00000268695:10 ENST00000269554:8 ENST00000270225:5 ENST00000271854:15 ENST00000275560:5 ENST00000276440:18 ENST00000281017:3 ENST00000281172:6 ENST00000286332:5 ENST00000291547:6 ENST00000292246:5 ENST00000293590:10 ENST00000294618:4 ENST00000295294:2 ENST00000295408:5 ENST00000296046:2 ENST00000296794:34 ENST00000296932:4 ENST00000297404:2 ENST00000298527:2 ENST00000301336:4 ENST00000302174:7 ENST00000306317:7 ENST00000306821:6 ENST00000307092:4 ENST00000309930:7 ENST00000310018:2 ENST00000312584:2 ENST00000312655:2 ENST00000314607:11 ENST00000322349:22 ENST00000322893:6 ENST00000333479:34 ENST00000333850:8 ENST00000335968:15 ENST00000335968:17 ENST00000336148:14 ENST00000342111:2 ENST00000342711:11 ENST00000343516:15 ENST00000344799:7 ENST00000345125:2 ENST00000348962:6 ENST00000349874:9 ENST00000350221:8 ENST00000352115:7 ENST00000352896:14 ENST00000354381:3 ENST00000355697:13 ENST00000355758:2 ENST00000356865:9 ENST00000357041:15 ENST00000357943:9 ENST00000358552:3 ENST00000358866:2 ENST00000359169:2 ENST00000359495:2 ENST00000360003:15 ENST00000360064:20 ENST00000360790:17 ENST00000360825:3 ENST00000361731:7 ENST00000361924:17 ENST00000366652:2 ENST00000366829:5 ENST00000367492:100 ENST00000367492:49 ENST00000367714:2 ENST00000368125:2 ENST00000368169:4 ENST00000368416:3 ENST00000368761:4 ENST00000369085:3 ENST00000369538:7 ENST00000370296:7 ENST00000370336:11 ENST00000370449:10 ENST00000371494:6 ENST00000371923:6 ENST00000372044:9 ENST00000374324:8 ENST00000375180:3 ENST00000375755:5 ENST00000377314:8 ENST00000377882:6 ENST00000379922:10 ENST00000380550:20 ENST00000380968:4 ENST00000380993:22 ENST00000381480:9 ENST00000381913:14 ENST00000382040:4 ENST00000382233:8 ENST00000383702:12 ENST00000383807:9 ENST00000388781:13 ENST00000389374:10 ENST00000389501:10 ENST00000389934:8 ENST00000390015:20 ENST00000391986:5 ENST00000392200:16 ENST00000393853:6 ENST00000394809:9 ENST00000395314:13 ENST00000395834:3 ENST00000395896:8 ENST00000396212:5 ENST00000396231:2 ENST00000397544:5 ENST00000397843:3 ENST00000397937:2 ENST00000398052:5 ENST00000398835:4 ENST00000399039:3 ENST00000404451:3 ENST00000404826:10 ENST00000406091:22 ENST00000406432:8 ENST00000406964:3 ENST00000410001:16 ENST00000412036:11 ENST00000412964:6 ENST00000414624:3 ENST00000415253:2 ENST00000416757:2 ENST00000417700:2 ENST00000418664:12 ENST00000421307:12 ENST00000421333:6 ENST00000422516:7 ENST00000423070:3 ENST00000423218:14 ENST00000424988:3 ENST00000426726:10 ENST00000427976:3 ENST00000430906:10

ENST00000431798:2 ENST00000432176:10
ENST00000434918:26 ENST00000435584:4
ENST00000435834:3 ENST00000437283:3
ENST00000438793:15 ENST00000439988:7
ENST00000440705:3 ENST00000443968:9
ENST00000446658:2 ENST00000446903:13
ENST00000447096:4 ENST00000447727:11
ENST00000450441:3 ENST00000452134:11
ENST00000452512:2 ENST00000452564:14
ENST00000453408:9 ENST00000454345:2
ENST00000454536:4 ENST00000457316:4
ENST00000460663:6 ENST00000461039:3
ENST00000463743:29 ENST00000464854:2
ENST00000470360:9 ENST00000470597:4
ENST00000480294:15 ENST00000480387:2
ENST00000480648:7 ENST00000483465:11
ENST00000483720:2 ENST00000484327:12
ENST00000488991:5 ENST00000489796:2
ENST00000490550:3 ENST00000491910:3
ENST00000492039:4 ENST00000492917:4
ENST00000493649:4 ENST00000493991:5
ENST00000507625:7 ENST00000510263:18
ENST00000512840:7 ENST00000515201:4
ENST00000515606:7 ENST00000518236:4
ENST00000518467:2 ENST00000519543:11
ENST00000522006:6 ENST00000522335:4
ENST00000522795:2 ENST00000524842:4
ENST00000529433:3 ENST00000531532:4
ENST00000532494:3 ENST00000533706:20
ENST00000533721:2 ENST00000535878:5
ENST00000536246:8 ENST00000536298:18
ENST00000537463:23 ENST00000537652:28
ENST00000538066:9 ENST00000538729:6
ENST00000539050:5 ENST00000539099:6
ENST00000539100:2 ENST00000539625:2
ENST00000540501:3 ENST00000542398:7
ENST00000542445:22 ENST00000542835:4
ENST00000542895:24 ENST00000544037:6
ENST00000544640:2 ENST00000544752:11
ENST00000544950:19 ENST00000545140:2
ENST00000546060:4 ENST00000546169:3
ENST00000548670:2 ENST00000550053:10
ENST00000550286:12 ENST00000552994:32
ENST00000553532:5 ENST00000554017:9
ENST00000554922:3 ENST00000556842:3
ENST00000558879:2 ENST00000559982:8
ENST00000561425:2 ENST00000568579:8

References

[1] Pickrell J.K., Marioni J.C., Pai A.A.,Degner J.F., Engelhardt B.E., Nkadori E., Veyrieras J.B., Stephens M. and Gilad Y. and Pritchard J.K. “Understanding mechanisms underlying human gene expression variation with RNA sequencing”, *Nature* 2010, 464:7289, 768–772.

[2] Arneet L. Saltzman, Qun Pan and Benjamin J. Blencowe “Regulation of alternative splicing by the core spliceosomal machinery”, *Genes Dev.* 2011, 25:373-384.
